# Mathematical Modeling of Influenza Dynamics: Integrating Seasonality and Gradual Waning Immunity

**DOI:** 10.1007/s11538-025-01454-w

**Published:** 2025-05-16

**Authors:** Carlos Andreu-Vilarroig, Gilberto González-Parra, Rafael-Jacinto Villanueva

**Affiliations:** 1 Instituto de Matemática Multidisciplinar, Universitat Politècnica de València, Camí de Vera, s/n, Valencia, 46022 Spain; 2 Department of Mathematics, New Mexico Tech, 801 Leroy Pl, Socorro, 87801 New Mexico USA

**Keywords:** Mathematical modeling, Influenza, Seasonality, Gradual waning immunity, Susceptibility, 92D30, 92-10

## Abstract

The dynamics of influenza virus spread is one of the most complex to model due to two crucial factors involved: seasonality and immunity. These factors have been typically addressed separately in mathematical modeling in epidemiology. In this paper, we present a mathematical modeling approach to consider simultaneously both forced-seasonality and gradual waning immunity. A seasonal SIR*n* model that integrates seasonality and gradual waning immunity is constructed. Seasonality has been modeled classically, by defining the transmission rate as a periodic function, with higher values in winter seasons. The progressive decline of immunity after infection has been introduced into the model structure by considering multiple recovered subpopulations or recovery states with transmission rates attenuated by a susceptibility factor that varies with the age of infection. To show the applicability of the proposed mathematical modeling approach to a real-world scenario, we have carried out a calibration of the model with the data series of influenza infections reported in the 2010-2020 period at the General Hospital of Castellón de la Plana, Spain. The results of the case study show the feasibility of the mathematical approach. We provide a discussion of the main features and insights of the proposed mathematical modeling approach presented in this study.

## Introduction

Influenza is one of the most prevalent and recurrent diseases worldwide. Due to its seasonal nature, around one billion cases are affected annually, including 3-5 million cases of severe illness, and it causes from $290\ 000$ to $650\ 000$ deaths due to its severe damage to the respiratory system [1, (Lofgren et al. 2007; Neumann and Kawaoka 2022; Tang and Loh 2016)]. It is known that this disease is caused by different strains of the influenza virus. However, from an epidemiological point of view, the transmission dynamics of the influenza virus occur by multiple factors, which makes its modeling and study very challenging (World Health Organization 2024; Basile et al. 2018; Brugger and Althaus 2020; Endo et al. 2021; Hill et al. 2019; Moorthy et al. 2012; Sara et al. 2020). Thus, despite many studies devoted to investigating the influenza dynamics in humans the understanding of it remains incomplete (Chen et al. 2022; Dalziel et al. 2018; Kenah et al. 2011; Magal et al. 2020; Moorthy et al. 2012; Tamerius et al. 2011; Zhang 2015).

One of the factors that explain influenza seasonal behavior every year is the high mutation capacity of the influenza virus (Liu and Walker 2023; Neher et al. 2016; Shi et al. 2010). Due to its viral RNA structure, which is more unstable and easily mutable than DNA, and its ability to colonize zoonotic reservoirs, which allows influenza to persist throughout the year in the population, influenza generates new variants every year (Webster and Govorkova 2014; Barr and Fearns 2016). This means that they are able to evade naturally acquired immunity or vaccines (i.e., immuno-escape) and maintain their infectious capacity in the human population. We also know that influenza is especially prevalent in winter seasons, where cold and humidity are factors that facilitate the infection (Lowen and Steel 2014). For instance, recently it has been found that the antiviral immune responses are weakened by cold exposure (Huang et al. 2023). The explanation of this is that cold exposure decreases the total extracellular vesicle secretion as well as diminishes microRNA packaging and antiviral binding affinity of individual extracellular vesicles. In addition, some viruses replicate better at lower temperatures than at the natural body temperature (Papadopoulos et al. 1999). There are other works that have found underlying molecular mechanisms that show that cold temperature may diminish the host’s innate immune response to viral infections (Foxman et al. 2015, 2016).

Another highly influential factor in seasonal influenza epidemic waves and their magnitude is the immunity against the virus. When a person is exposed to a virus and develops an immune response, this person acquires immunological memory (Combadière et al. 2010; Cox et al. 2004; Kim et al. 2018; Olmos Liceaga et al. 2023). It is well-known that immunity varies depending on many factors including the host immune response of each individual and the time since the last infection, which is related to the waning effect of immunity (Krammer 2019). It is known that the host immune response affects the severity and duration of influenza infection or other type of virus infections (Barrett et al. 2021; Dobrovolny et al. 2013). Moreover, the susceptibility can vary depending on the virus strain to which the host is exposed, but also from past exposures to the same or similar strains, which may confer the so-called cross-immunity. (Ahmed et al. 2007; Barrett et al. 2021; Cobey and Hensley 2017).

A large part of the population acquires immunity through having been infected, while another part of the population acquires it thanks to the vaccine, as a preventive action to be protected against the virus. It is generally known that natural immunity lasts longer than vaccine immunity and protects against more types of strains (thanks to the cross-immunity) (Epstein and Price 2010; Krammer 2019). Regarding the effects of vaccination immunity on influenza transmission, only some part of the population is directly protected by vaccination immunity due to vaccination coverage. This coverage depends on many variables such as the vaccine’s economic cost and availability, vaccination campaign strategies, the population’s willingness to be vaccinated, etc. (Tracht et al. 2010; Arda et al. 2011; Castillo-Rodríguez et al. 2022; Imai et al. 2018; Putri et al. 2018). Additionally, it should be noted that the vaccine’s ability to confer immunity (its efficacy) is usually partial, and not necessarily against all circulating virus strains. In contrast, the natural immune response has several components and mechanisms of action that are not yet very well understood (Krammer 2019; Patel et al. 2021). The immune response can be divided into two main pieces; the adaptive and the innate response. A key property of the adaptive response is its memory (Kubo et al. 2017; Ratajczak et al. 2018). This memory helps to fight viral infections since it mounts a quick and specific response that recognizes an antigen that the body has previously encountered (Natoli and Ostuni 2019; Ratajczak et al. 2018). It has been mentioned that the immune memory plays an important role in the severity of an influenza infection (Azambuja 2010; Doherty et al. 2006). Cross-immunity is strongly related to the memory immune response which mainly originates from previous exposure to different viruses, and it plays an important role in repeated infections, pathogen diversity and mutations (Abu-Raddad and Ferguson 2004). As a particular case of the cross-immunity, during the well-known Spanish influenza pandemic of 1918, there were more than 17 million deaths (Ahmed et al. 2007; Noymer and Garenne 2000). Interestingly, 30-60 years old adults presented a lower mortality rate in comparison with younger adults aged 18-30 years. It has been argued that this happened due to factors related to the immunological memory (Ahmed et al. 2007; Gagnon et al. 2013, 2015; McAuley et al. 2015).

Based on all the previous epidemiological aspects, the mathematical modeling of seasonal influenza transmission dynamics is challenging and complex, especially if both seasonality or immunity factor are to be taken into account. Different mathematical models have been presented to study the dynamics of influenza epidemics and pandemics considering these two key factors, although the most widespread has been the compartmental models based on differential equations (Brauer et al. 2012; Yuan et al. 2021).

On the one hand, some mathematical models for influenza include a seasonally-forced function – classically, a time-dependent sinusoidal infection rate with a 1-year period – in order to generate a seasonal behavior in the infected population (Gabrick et al. 2024; Towers and Feng 2009). In Truscott et al. (2012), a model with a seasonally-forced function was used to estimate that a seasonal peak basic reproduction number is in the range of 1.6-3. In addition, it was suggested that the basic reproduction number depends on the timescale for the waning immunity to the circulating influenza strain, which was estimated to be between 3 and 8 years. Also, it was found that seasonal variation in transmissibility is in the range of 15-30% of its mean value. Another recent work that deals with seasonal influenza and the estimation of the basic reproduction number is presented in Jing et al. (2020). The authors used a seasonal forced function and took into account meteorological factors including unreported cases. Nevertheless, seasonal behavior can also occur without a seasonally-forced function (Hethcote et al. 1981).

On the other hand, in compartmental models, immunity decay has been modeled by modifying the classical model equations into integro-differential equations (Bhattacharya and Adler 2012). The well-known SIR and SEIR epidemic model structures have been used to model waning immunity with regard to the influenza virus (Ehrhardt et al. 2019; Takeuchi and Kuroda 2010). Previous works have studied influenza by means of a variety of mathematical models that include gradual waning immunity explicitly. All these works provide important insights into the dynamics of seasonal influenza. For instance, in Goeyvaerts et al. (2015), a SEIRS mathematical model with age stratified classes and vaccination was presented. Each year some proportion of the susceptible population is seeded into the population as newly infectious individuals and a seasonal forced function was used. Despite some problems with model identifiability due to the correlation of several parameters, it was highlighted the importance of influenza seasonal variation. In El Khalifi and Britton (2023); Hethcote et al. (1981); Guiaş (2023), the authors divide the susceptible population into multiple stages with different immunity levels in order to simulate the immunity decay after infection. In Hethcote et al. (1981), a mathematical model that considers multiple recovered classes, but with the possibility of reinfection only in the last recovered class is constructed and analyzed. In El Khalifi and Britton (2023), a mathematical model that considers several recovered stages but no forced-seasonality in the transmission rate has been presented.

Regarding mathematical models for influenza that have included forced seasonality and explicit gradual waning immunity together, there are few works. In Dafilis et al. (2014), two recovered classes are included in order to consider gradual waning immunity but in the last recovered class people can transit to the completely susceptible population. In Xiao and Moghadas (2013), a mathematical model that includes impulse vaccination (even distribution) before each season was presented. The main simulation result was that the pattern of large seasonal epidemics is strongly correlated with the duration of specific cross-protection immunity induced by natural infection. The possibility of moving from the last recovered class to susceptible is considered in the model. Their study included a large range of simulations to explore the effect of several parameters. In Lai and Gog (2024), a new model based on a discrete system was presented in order to show that the waning immunity can produce sustained oscillations without using any seasonally-forced function.

In this paper, we propose a mathematical modeling approach to describe the dynamics of seasonal influenza combining forced-seasonality and gradual waning immunity. The proposed compartmental model – the seasonal SIR*n* model – considers that the population that have been infected will transit through multiple recovered stages. In each stage, the individuals have a different susceptibility (or immunity) level, which modulates the infection rate of the individuals in the recovered stages. In particular, the susceptibility level (and thus, the probability of infection) increases as individuals progress through the recovered stages. In addition, the mathematical modeling approach takes into account the seasonality of the influenza virus by considering the infection rate as a 1-year periodic sinusoidal function. Some previous models (Hethcote et al. 1981; El Khalifi and Britton 2023) can be seen as good foundations for the model presented in this work. In order to show the applicability and reliability of the constructed mathematical modeling approach, we consider a case study by using the data series of influenza infections reported in the 2010-2020 period at the General Hospital of Castellón de la Plana city, Spain. We carry out a calibration process to fit the seasonal SIR*n* model to the real data. In addition, a brief practical identifiability analysis of the unknown model’s parameters is conducted for the case study.

The paper is structured as follows. In Section 2 we present in detail a complete description of the seasonal influenza mathematical model, including a biological interpretation of its equations and parameters. Section 3 is devoted to show the applicability and reliability of the proposed mathematical modeling approach by presenting a real-world case study. Finally, discussion and conclusions are presented in Sections 4 and 5, respectively.

## Mathematical model

In this section, we present the foundation and main features of the proposed mathematical modeling approach that couples seasonality and immunity in influenza epidemiological dynamics. In particular, we present the seasonal influenza mathematical model, including a description of its equations and parameters. First, let us proceed to present the seasonal SIR*n* model.

### Mathematical model description

**Fig. 1:**
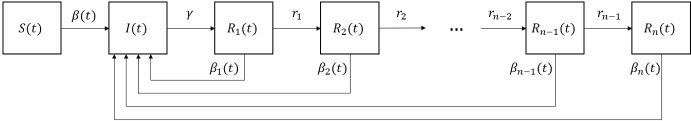
Model flow chart of the seasonal SIR*n* influenza epidemic model

The constructed mathematical model, which we called the seasonal SIR*n* model, assumes that the entire population *N*(*t*) at time *t* is divided into $n + 2$ subpopulations: the susceptible *S*(*t*), the infectious *I*(*t*) and the *n* consecutive recovered $R_1(t),\dots , R_n(t)$, where $n \in \mathbb {N},\ n \ge 2$. In contrast to the classical epidemiological models, where only one population of recovered individuals *R*(*t*) appears, this model divides the recovered individuals into multiple stages according to their immunity level, which depends on the age of infection. The transitions between the multiple recovered stages enable the immunity progression of the individuals. The mathematical model schema is shown in Figure 1.

The natural process of infection due to influenza virus is the following:

1. A susceptible individual becomes infected due to a contact with other infected individuals. The decrease in susceptibles *S*(*t*), or equivalently, the increase of infecteds *I*(*t*) over time due to contact transmission is proportional to the number of possible contacts between susceptibles and infected *S*(*t*)*I*(*t*).
2. The transmission rate $\beta =\beta (t)$ determines what fraction of all possible contacts are effective contacts, resulting in infection, and addresses all factors affecting contagion.
3. An infected individual becomes recovered after an infectious period of $\frac{1}{\gamma }$ days. It is estimated that the influenza infection time is between 5 and 7 days [69]. In this study, we assume $\gamma = 1/7\ \text {days}^{-1} = 1\ \text {weeks}^{-1}$.
4. A recovered person, immediately after infection, has some level of susceptibility to the virus. However, as time passes after infection, he/she progressively loses immunity. Approximating this phenomenon by a discrete process, this individual will transition through *n* recovered stages, each with a lower immunity level. The time in which the individual is in an $R_k$ recovered stage before moving to the $R_{k+1}$ stage is $\frac{1}{r_k}$ days. The value of $r_k$ depends on how many and in what form the recovery stages are defined. Additionally, in each of these $R_k$ recovered stages, there is a possibility that individuals will be re-infected by contact with infected individuals. Thus, the decrease of individuals in the recovered stage $R_k(t)$ is proportional to the number of contacts between recovered and infected $R_k(t) I(t)$, with a transmission rate $\beta _k(t)/N$, which depends on the contagion factors, and on the immunity level of the recovered individuals.
5. All populations are affected by total mortality (i.e. natural and infection-induced mortality), which occurs proportionally to the size of each population with a constant death rate $\mu$. In contrast, births occur only in the susceptible population, since we assume that all those born do not have immunity to the influenza virus. We also assume that newborns are proportional to the entire population *N*(*t*) with a constant birth rate $\lambda$ (Hethcote 2000).

The mathematical model is given by the following non-autonomous system of nonlinear first-order ordinary differential equations:

1 $$\begin{aligned} {\left\{ \begin{array}{ll} \dot{S}(t) & = \lambda N(t) - \mu S(t) - \beta (t) \frac{S(t) I(t)}{N(t)}, \\ \dot{I}(t) & = \beta (t) \frac{S(t) I(t)}{N(t)} + \sum _{k=1}^n \beta _k(t) \frac{R_k(t) I(t)}{N(t)} - \gamma I(t) - \mu I(t), \\ \dot{R_1}(t) & = \gamma I(t) - \beta _1(t) \frac{R_1(t) I(t)}{N(t)} - r_1 R_1(t) - \mu R_1(t), \\ \vdots \\ \dot{R_k}(t) & = r_{k-1} R_{k-1}(t) - \beta _k(t) \frac{R_k(t) I(t)}{N(t)} - r_k R_k(t) - \mu R_k(t),\ \forall k=2,\dots ,n-1 \\ \vdots \\ \dot{R_n}(t) & = r_{n-1} R_{n-1}(t) - \beta _n(t) \frac{R_n(t) I(t)}{N(t)} - \mu R_n(t), \\ \dot{N}(t) & = \dot{S}(t) + \dot{I}(t) + \sum _{k=1}^n \dot{R}_k(t) \quad \equiv \quad \dot{N}(t)= (\lambda - \mu ) N(t), \end{array}\right. } \end{aligned}$$

with $S(0), I(0), R_k(0), N(0) \ge 0,\ \forall k=1,\dots ,n,$ as initial conditions, and where $\lambda \in \mathbb {R}_+$ is the birth rate, $\mu \in \mathbb {R}_+$ is the death rate, $\gamma \in \mathbb {R}_+$ is the recovery rate, $\beta (t):\mathbb {R}_+ \rightarrow \mathbb {R}_+$ is the transmission rate at time *t* for the *S* susceptible subpopulation, $\beta _k(t):\mathbb {R}_+ \rightarrow \mathbb {R}_+,\ k=1,\dots ,n$ is the transmission rate at time *t* for the $R_k$ recovered subpopulation, and $r_k \in \mathbb {R}_+,\ k=1,\dots ,n-1$ is the transition rate between $R_k$ and $R_{k+1}$ recovered subpopulations due to the waning immunity of the recovered individuals.

### Immunity and susceptibility

As it has been previously explained, people who have been exposed to the influenza virus acquire a memory in their immune system, also known as natural immunity. However, this immunity is progressively lost or is partial against other strains of the virus. Thus, the individual becomes less immune, or in other words, more susceptible to becoming re-infected with the virus. In this study, in order to analyze the dynamics of seasonal influenza at the human population level, we consider that the infection of individuals can occur against all existing circulating variants, without distinguishing between them in the proposed model. Although the predominance of one strain over another may indeed change between seasons (due to the mutation of the virus and also to the progressively loss of immunity), this predominance alternates in a complex way between strains. Thus, our interest lies in modeling the immunity of individuals against all these strains on average and in the medium to the long term.

Additionally, an important aspect to consider is the effect of acquired immunity from vaccination. Individuals vaccinated in previous years are distributed in the recovered classes $R_k(t)$, as they have a certain level of immunity that varies according to the circulating influenza variants (Chan et al. 2018; Furuse and Oshitani 2016; Li et al. 2016). Explicit modelling of vaccination has been introduced in similar models by allowing transitions (connections) between recovered classes $R_k(t)$, allowing an individual to regain higher levels of immunity upon vaccination (El Khalifi and Britton 2023). In our case, as we are working with a more simplified model (since we do not have vaccination data), the effect of vaccine-induced immunity will be captured by the model parameters in the calibration process. In particular, vaccine immunity will result in a lower value of the beta infection rate, because the vaccinated population hinders the transmission and spread of the virus in the overall population.

The constructed SIR*n* model captures the immunity effect introducing multiple recovered stages $R_k(t),\ k=1,\dots ,n$, according to the individuals’ immunity/susceptibility level. Here, we define the susceptibility level $s(\tau ): \mathbb {R}_+ \rightarrow [0,1]$ as the relative ability of an individual to become infected at a time after infection $\tau$ with respect to a fully susceptible individual. It is important to note that the time after infection $\tau$ is different and independent from the time *t* of the model. The susceptibility function $s(\tau )$ is bounded between 0 (not susceptible/full immunized) and 1 (fully susceptible or, equivalently, not immunized). By this definition, immunity can be defined as $i(\tau ) = 1 - s(\tau )$. Because of the natural immunity decay, it is reasonable to define $i(\tau )$ as a monotonic decreasing function and, consequently, $s(\tau )$ as a monotonic increasing function (Ranjeva et al. 2019).

Since immunity decay is a continuous process over time $\tau$, there are infinite phases of susceptibility, and therefore, of recovered stages. It follows that, in order to design a compartmental model such as the one proposed here, we have chosen a partition

$$\mathcal {T} = \{\tau _1,\dots ,\tau _n\}$$

of different $\tau _i$ times in the $R_+ = [0,+\infty )$ interval, so that we obtain a set of susceptibility levels

$$\mathcal {S} = \{s(\tau _1),\dots ,s(\tau _n)\}.$$

The partition can be arbitrarily chosen, although it is essential that it be sufficiently refined to faithfully represent the susceptibility increase process given by the $s(\tau )$ susceptibility function shape. In this way, the *n* recovered subpopulations or states $R_k(t)$ are defined, each with its level of susceptibility $s(\tau _k),\ \forall k=1,\dots ,n$. The *n* parameter is similar to the number of stages used in the gamma distributed epidemiological models, and gives to the recovered phase a more realistic distribution than the classical exponential distribution that is oftentimes used in epidemiological models (Best and Perelson 2018; González-Parra et al. 2018; Lloyd 2001; O’Neill and Becker 2001). Alternatively to this approach, an integro-differential system can be constructed using a continuous susceptibility function $s(\tau )$ (Hethcote et al. 1981; Hethcote 2000). In Brauer et al. (2019) it has been mentioned that it is possible to assume that at the beginning of a seasonal influenza season individuals have some level of cross-immunity that depends on the previous seasonal influenza strains, up to a maximum of *n* seasons as we have assumed in the proposed seasonal SIR*n* model.

### Transition and transmission rates

Having defined the partition $\mathcal {T}$, the transition rate $r_k$ between the $R_{k-1}(t)$ and $R_k(t)$ recovered subpopulations can be defined as

2 $$\begin{aligned} r_k = \frac{1}{\Delta \tau _k} = \frac{1}{\tau _{k+1} - \tau _k},\quad \forall k=1,\dots ,n-1. \end{aligned}$$

Note that these transition rates are equal if the partition $\mathcal {T}$ is uniformly divided.

Regarding the transmission rates, $\beta (t)$ and $\beta _k(t)$, these are related, as in classical models, with the effective contact rates between individuals (with the probability of an individual becoming infectious after an effective contact), but also with the susceptibility level of the individuals. Using the susceptibility function, the transmission rate $\beta _k(t)$ for the $R_k(t)$ recovered subpopulation can be defined as

3 $$\begin{aligned} \beta _k(t) = s(\tau _k) \beta (t) = [1 - i(\tau _k)] \, \beta (t), \quad k = 1,\dots ,n. \end{aligned}$$

With this definition, note that, if the susceptibility function $s(\tau )$ is monotonically decreasing, then one gets that $\beta _k < \beta _{k+1},\ \forall k=1,\dots ,n-1$. As particular cases of interest,

- Typically, $\tau _1 = 0$ and $s(\tau _1) = s(0) \simeq 0$, so that $\beta _1 = s(0) \beta (t) = 0$, i.e., individuals after infection in $R_1$ population are completely immunized and cannot be infected ($R_1$ is the classical *R* recovered population).
- If $s(\tau _n) \simeq 1$, then $\beta _n = s(\tau _n) \beta (t) \simeq \beta (t)$, i.e., after a long period of time following infection, individuals in $R_n$ population are almost completely susceptible to reinfection.

Thus, based on the previous aspects to fully characterize the influenza infection dynamics with the seasonal SIR*n* model, it is sufficient to define $\gamma$, $\beta (t)$, $s(\tau )$ (or alternatively, $i(\tau )$), and the partition $\mathcal {T}$.

### Seasonality

With regard to the seasonality, we know that there are infections that depend on the season of the year, since there are several factors at that season (temperature, humidity, contacts, human behavior, etc.) that contribute to the spread of the pathogens (Foxman et al. 2015, 2016; Huang et al. 2023; Lowen and Steel 2014; Papadopoulos et al. 1999). Usually, the winter seasons are those in which seasonal influenza waves occur (Ewing et al. 2017; Greiff et al. 2021; González-Parra et al. 2011; Hill et al. 2019; Ho et al. 2019). One of the classic ways of modeling seasonality in epidemiological mathematical models is to define the transmission rate as a time-dependent function $\beta (t)$ (Arenas et al. 2021; Grassly and Fraser 2006; Jing et al. 2020; Tanaka and Aihara 2013). If the transmission rate were to be considered constant and only one recovered stage is considered, then the theoretical analysis would show that this kind of models has only a global stable disease-free and endemic equilibrium points (Hethcote et al. 1981; Stech and Williams 1981; El Khalifi and Britton 2023; Guiaş 2023), which does not reflect the seasonal character of the influenza disease. Some exceptions to this situation are some models based on a delay differential equation, where periodic solutions can be obtained (Hethcote 2000), and network models (Acedo et al. 2011), where seasonality arises in a natural way because of the network structure.

In our model, it is clear from Eq. (3) that the transmission rates $\beta _k(t)$ depend upon $\beta (t)$. Based on epidemiological aspects related to seasonal influenza (Ewing et al. 2017; Huang et al. 2023), we propose in this study that $\beta (t)$ should be a sinusoidal periodic function. Its expression is given by

4 $$\begin{aligned} \beta (t) = \frac{\beta _0}{2} \left[ 1 + \cos \left( \frac{2\pi }{T} t - \phi _0\right) \right] \in [0,\beta _0], \end{aligned}$$

where $\beta _0$ is the maximum value of the transmission rate, $\phi _0$ is the initial phase, and *T* is the seasonal period, which, based on epidemiological and biological findings, should be around one year (Huang et al. 2023; Lowen and Steel 2014; Thai et al. 2015).

## Case study: seasonal influenza in Castellón de la Plana city, Valencian Community, Spain

In this section, we use the proposed mathematical modeling approach to describe a real-world case study to explore the applicability and feasibility of the seasonal SIR*n* model. The case study corresponds to the seasonal influenza between the years 2011 and 2020 in Castellón de la Plana city, in the Valencian Community, Spain. To test the model’s ability to represent this scenario, we will fit the model to the available data through a calibration process. It is important to remark that the main motivation of the presentation of the case study is to show the applicability and feasibility of the seasonal SIR*n* model to a real-world situation instead of showing a calibration process to estimate the parameters of the seasonal SIR*n* model given a real data of influenza. For instance, in Goeyvaerts et al. (2015) a more complex model was fitted to real data and it was found that the model was unidentifiable. Let us proceed to present the case study.

### Data and known parameters

As available calibration data, we have the weekly influenza urgent reported cases in the Castellón de la Plana General Hospital between 2010 and 2020 years, $\textbf{I}_r = \left\{ I_r^{t_i}\right\} _{i=0}^m$, where $t_0 = 0$ represents the start date, August 9th, 2010 (week 32), and where $t_m = t_{507} = 507$ (weeks) represents the end date, April 27th, 2020 (week 18). It is important to mention that there are available data series of previous years. However, the 2009-2010 season had the entry of a new influenza strain: the A/H1N1 variant (González-Parra et al. 2011; Shubin et al. 2016). In this season, there was a highly atypical epidemic wave, due to a lower level of immunity in the population. In this work, the seasonal SIR*n* model has been oriented to medium- to long-term influenza disease, with multiple strains already established in the population and against which the population has acquired a certain immunity. In this case, the alternating circulation of the strains makes that, on average, the magnitude of influenza waves periodically reaches a certain “seasonal steady state”. This can be seen in the height of the peaks of reported cases, which are usually similar in different seasons. For this reason, we have chosen a period such as 2010-2020, in which the magnitude of the flu epidemic has been observed regular.

**Fig. 2:**
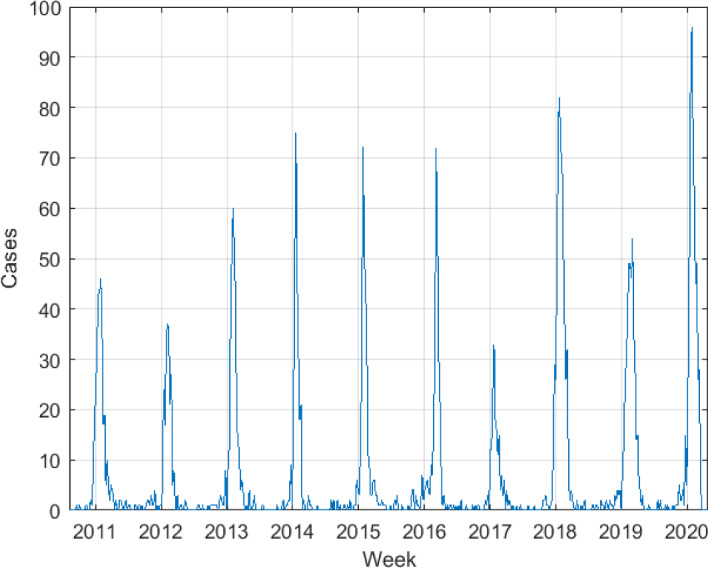
Seasonal influenza data series: weekly influenza urgent reported cases in the Castellón de la Plana General Hospital in the 2010-2020 period. Source: Clinical Documentation and Admissions Department of the Castellón de la Plana General Hospital (private data)

The seasonal influenza data series are shown in Figure 2.

The entire population of Castellón de la Plana is covered by this hospital, so the cases reported are really those of the city in the selected period (private data from the Clinical Documentation and Admissions Department of the Castellón de la Plana General Hospital). Specifically, we know that

- The average annual birth rate in the 2014-2019 period (previous years were not available) was 8.433 births per 1000 inhabitants, i.e., $\lambda = 8.433/(1000 \cdot 52) = 1.623 \cdot 10^{-4} \text { weeks}^{-1}$ [91]. The average annual death rate in the same period was 8.435 deaths per 1000 inhabitants, i.e., $\mu = 8.435/(1000 \cdot 52) = 1.622 \cdot 10^{-4} \text { weeks}^{-1}$. With these values, we can assume that the rates are equal and fixed, taking the average, that $\lambda = \mu = 1.622 \cdot 10^{-4} \text { weeks}^{-1}$. And consequently, we can assume that the size of the total population $N(t) = N$ is constant.
- The average total population size assigned to the hospital is $N = 282\ 967$, according to the information we have received from the Clinical Documentation and Admissions Department of the hospital (private communication from Clinical Documentation and Admissions Department of the Castellón de la Plana General Hospital). Thus, *N* represents the number of individuals in the wider community who would attend the hospital if they became severely ill.

In addition, we use crucial information (or constraint) for the calibration process: it has been estimated that the number of people who end up getting infected by seasonal influenza each year is between 5 and 15% of the total population [92, (Russell et al. 2008; Stöhr 2002; Tokars et al. 2018)].

Figure 2 shows that every year there is a peak and only one wave per year. Moreover, as expected, this peak occurs over the winter season, although its height varies due to the intrinsic randomness of the virus infection process, its severity on infected individuals and circulating influenza strains (Goeyvaerts et al. 2015). This qualitative behavior is also observed in many other countries around the world (Truscott et al. 2012; Hirve et al. 2016; World Health Organization 2024; Yuan et al. 2021). Using the peak information in the seasonal influenza data series, we have obtained the period *T* and the initial phase $\phi _0$ of the $\beta (t)$ function. First, we have detected the date when the peaks of each season occur, and we have calculated that the average distance between peaks is $T = 52.43 \text { weeks},$ i.e., approximately one year, as we already expected. Then, to provide a congruent value of the initial phase $\phi _0$, we have fixed that the transmission rate $\beta (t)$ reaches its maximum (i.e., $\beta (t) = \beta _0$) around the peaks of the seasons, and its minimum (i.e., $\beta (t) = 0$) just at the midpoint between seasons. Therefore, we have taken all the dates located just at the midpoint between two seasons, and we calculated that, on average, the inter-season date (with minimum transmission rate) is around August 6th. Since our series starts on August 9th (practically on the same date, with only three days of difference), we set $\beta (t_0) = \beta (0) = 0$, and conclude that

$$\begin{aligned} \beta (0) = \frac{\beta _0}{2} \left[ 1 + \cos \left( -\phi _0\right) \right] = 0 \quad \Longrightarrow \quad \phi _0 = \arccos (-1) = \pi . \end{aligned}$$

Therefore, for our case study, we define $\beta (t)$ as

5 $$\begin{aligned} \beta (t) = \frac{\beta _0}{2} \left[ 1 + \cos \left( \frac{2\pi }{52.43} - \pi \right) \right] . \end{aligned}$$

For our case study we have proposed a susceptibility function $s(\tau )$ that has an exponential type, with the form

6 $$\begin{aligned} s(\tau )&= 1 - (1 - s_0) e^{-a\tau } \in [s_0,1), \quad \forall \tau \ge 0, \end{aligned}$$

where $s_0 \in (0,1]$ represents the initial susceptibility degree after infection and the exponential parameter $a \in \mathbb {R}_+$ modulates the increase in susceptibility, or equivalently, the immunity decay, over time after infection $\tau$. The larger the value of *a*, the faster the susceptibility increases. Complementary to this function, the immunity degree function can be defined as

7 $$\begin{aligned} i(\tau )&= 1 - s(\tau ) = (1 - s_0) e^{-a\tau } \in [0,1-s_0), \quad \forall \tau > 0. \end{aligned}$$

Some particular aspects and assumptions related to the chosen susceptibility function $s(\tau )$ are the following:

- Immediately after infection, the individual has an initial degree of susceptibility $s_0 \in (0,1]$, i.e. $s(0) = s_0$. This degree of susceptibility to a single virus strain of influenza could be set approximately to $s_0 \approx 0$, since immediately after infection, the individual is almost fully protected against this strain. Nevertheless, in the context of seasonal influenza, where there are multiple circulating strains, an individual can become infected by other variants after an infection. It should be taken into account that, after infection, the immune system is very active and there is cross-immunity between strains (El Khalifi and Britton 2023; Goeyvaerts et al. 2015; Xiao and Moghadas 2013). Thus, the susceptibility degree is assumed as $0 < s_0 \le 1$.
- $s(\tau ) \rightarrow 1$ when $\tau \rightarrow \infty$, i.e., as the time after infection increases, the individual tends to become completely susceptible (to lose all immunity). This is related to the fact that the protective immunity wanes and also to the fact that new strains appear which increases the likelihood of becoming infected again (Goeyvaerts et al. 2015; Xiao and Moghadas 2013; Andreasen and Sasaki 2006; De Cezaro et al. 2023; Omori and Sasaki 2013).

For this case study, we choose the value of *a* such that at 6 years after an individual becomes infected ($\tau =6$), i.e., 312 weeks after infection (considering 52 weeks per year), the susceptibility degree has recovered to 95% (Xiao and Moghadas 2013; Pitman et al. 2013; Gibson et al. 2016). Thus,

8 $$\begin{aligned} s(312) = 1 - (1 - s_0) e^{-a \cdot 312} = 0.95 \quad \Longrightarrow \quad a = - \frac{1}{312} \log \left( \frac{1 - 0.95}{1 - s_0}\right) . \end{aligned}$$

**Fig. 3:**
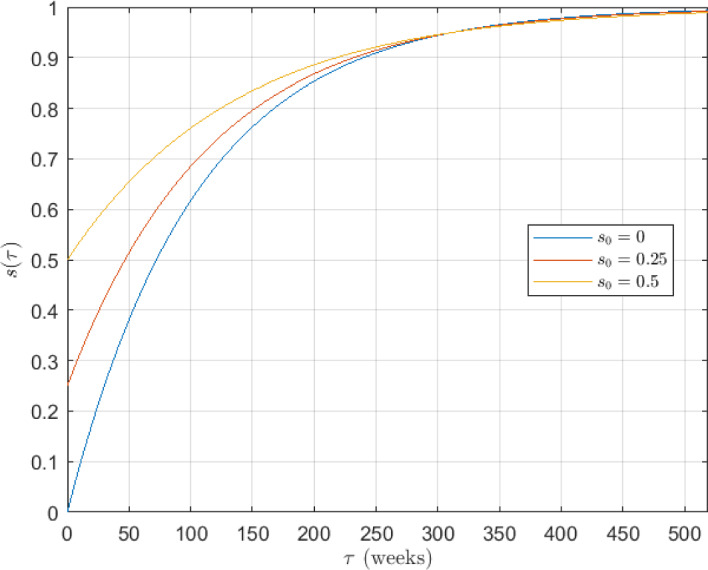
Susceptibility functions $s_i(\tau )$ for different initial susceptibility degrees $s_0$ and exponential parameter *a*

We will also consider three scenarios for the initial susceptibility degree $s_0$: 0%, 25% and 50%. The susceptibility functions $s_i(\tau )$ for this case study are shown in Figure 3, and are given by

9 $$\begin{aligned} s_0 = 0.00,\ a_1 = 9.602 \cdot 10^{-3} \quad&\Longrightarrow \quad s_1(\tau ) = 1 - e^{-9.602 \cdot 10^{-3} \tau }, \end{aligned}$$

10 $$\begin{aligned} s_0 = 0.25,\ a_2 = 8.680 \cdot 10^{-3} \quad&\Longrightarrow \quad s_2(\tau ) = 1 - 0.75\, e^{-8.680 \cdot 10^{-3} \tau }, \end{aligned}$$

11 $$\begin{aligned} s_0 = 0.50,\ a_3 = 7.380 \cdot 10^{-3} \quad&\Longrightarrow \quad s_3(\tau ) = 1 - 0.50\, e^{-7.380 \cdot 10^{-3} \tau }. \end{aligned}$$

The partition $\mathcal {T}$ has been applied over a sufficiently large recovery period: 10 years (520 weeks). For this period, we can guarantee that the immunity of a recovered individual would have been reduced to an extremely low level. As the temporal unit of the calibration data is the week, we have defined the partition

$$\mathcal {T} = \{\tau _1,\tau _2,\dots ,\tau _{520}\} = \{0,1,\dots ,519\},$$

with $n = 520$ recovered subpopulations, so that

$$\mathcal {S} = \{s(\tau _1),s(\tau _2),\dots ,s(\tau _n)\} = \{0,9.556 \cdot 10^{-3},\dots ,0.993\}$$

contains the susceptibility degree of each recovered subpopulation. In particular, note that when an individual arrives at the last recovered subpopulation, it has a susceptibility degree of $s(\tau _{520}) = 0.993 \simeq 1$ (is almost fully susceptible), or an immunity degree of $i(\tau _{520}) \simeq 0$.

With this equidistributed partition $\mathcal {T}$, we can compute the transition rates between the recovered states $R_k$ and $R_{k+1}$ as

$$r_k = \frac{1}{\tau _{k+1} - \tau _k} = 1,\quad \forall k=1,\dots ,n-1.$$

The parameters of model (1), with their description, units and values for the case study, are summarized in Table 1.

**Table 1:** Model parameters, with their description, units and values for the case study

**Parameter**	**Description**	**Units**	**Value**
$\beta (t)$	Transmission rate function	$\text {time units}^{-1}$	$\frac{\beta _0}{2} \left[ 1 + \cos \left( \frac{2\pi }{T} t - \phi _0\right) \right]$
$\quad \beta _0$	Amplitude (maximum value) of $\beta (t)$	$\text {time units}^{-1}$	Unknown
$\quad T$	Period of $\beta (t)$	$\text {time units}$	$52.43 \ \text {weeks}$
$\quad \phi _0$	Initial phase of $\beta (t)$	adimensional	$\pi$
$s(\tau _k)$	Susceptibility degree function	adimensional	$1 - e^{-9.602 \cdot 10^{-3} \tau _k}$
$r_k$	$R_k$ recovered transition rate	$\text {time units}^{-1}$	$1 \ \text {weeks}^{-1}$
$\gamma$	Recovery rate	$\text {time units}^{-1}$	$1 \ \text {weeks}^{-1}$
$\lambda$ and $\mu$	Birth and death rates	$\text {time units}^{-1}$	$1.622 \cdot 10^{-4} \ \text {weeks}^{-1}$

### Calibration process

In this subsection, we present the details related to the calibration process of the model to the seasonal influenza data as a real-world case study. The aim of the calibration is to find the estimated numerical values of the unknown model parameters set $\theta$ that best fits the available seasonal influenza data. This calibration process is relevant since it allows us to get insight into the seasonal SIR*n* model dynamics behavior and also to show the applicability of the seasonal SIR*n* model to the real-world.

In our case study, the unknown model parameter is the amplitude $\beta _0$ of the $\beta (t)$ function (see Table 1). The initial conditions of the problem are usually not known. Nevertheless, we are considering for calibration a period in which the magnitude of the epidemic has been similar in all seasons, i.e., it has reached a “seasonal steady state” (as mentioned in Sections 3.1 and 2.2). In order to reach this scenario in the model simulations, first we set the initial conditions to

$$S(0) = N - 1,\ I(0) = 1,\ R_1(0) = \dots = R_n(0) = 0.$$

Then, we numerically simulate the model for a long time (warm-up simulation period) until the model reaches a regular periodic behaviour and then obtain the initial conditions when the infected cases are minimal. In our case study, a 20 years warm-up period has been proved enough.

We implement the calibration process using the classical deterministic least square error minimization problem to find the optimal unknown parameters set $\theta ^*$ that best fits the model to the real-world seasonal influenza data. In our case, the data series is the weekly influenza urgent reported cases (Figure 2). However, it should be noted that this series cannot be directly compared with the infected individuals $I(t) = I(t;\beta _0)$ of the model (1), since this refers to the infected of the entire population, while the data series $\textbf{I}_r$ only counts the cases reported in the hospital. These cases are those individuals who have been most severely affected by the virus, and who have required hospital medical care. Therefore, we will assume that at week $t_i$, only a fraction *p* of the total cases are finally reported. Thus, the reported infected cases of the model can be expressed as

12 $$\begin{aligned} I_r(t_i) = p\, I(t_i),\ \forall i=1,\dots ,m, \end{aligned}$$

where $p \in [0,1]$ is the reporting fraction, which is also an unknown parameter. At this point, the unknown parameter set $\theta$ is defined as

13 $$\begin{aligned} \theta = (\beta _0,p) \in \Theta , \end{aligned}$$

where $\Theta = \mathbb {R}_+ \times [0,1]$ is the parameters’ search space. Then, the model calibration consists of solving the following least squares minimization problem:

14 $$\begin{aligned} \underset{\theta \in \Theta }{\text {minimize}} \sum _{i=1}^m \left( I_r(t_i) - I_{r}^{t_i} \right) ^2. \end{aligned}$$

It is important to point out that the data series represents the new weekly reported cases and *I*(*t*) is the number of influenza cases at time *t*. Since we assume that the average infectious period is 7 days, then *I*(*t*) is a good approximation of the new number of cases per week (Chowell 2017). Notice that *I*(*t*) is the concurrent infected individuals, which in average stay infectious one week.

As $I(t_i)$ is governed by a nonlinear differential equation, there is no analytical solution for the model (1), so the problem must be solved numerically. Based on the seasonal SIR*n* model and the minimization problem, we can apply two key points to the calibration process:

- Although the value provided for the initial phase $\phi _0 = \pi$ is a good approximation, it may not be optimal depending on the value of $\beta _0$. This is because $\beta _0$ has the effect of changing the infection force and, consequently, slightly advancing or delaying epidemic waves. Therefore, in order to give more flexibility to the model, we compute after each simulation the discrete cross-correlation function $(\textbf{I} \star \textbf{I}_r)(\lambda )$ between the series of total infected $\textbf{I} = \{I(t_i)\}_{i=1}^m$ and reported $\textbf{I}_r$ (Rabiner and Gold 1975; Teixeira et al. 2017). Since we know that $\phi _0 \simeq \pi$ is a phase very close to the real one, we have limited the lag $\lambda$ to 52 weeks (1 year), so that $\lambda = -52,\dots , 0, \dots , 52$. By doing so, we find the lag $\lambda _0$ that causes the maximum cross-correlation, i.e.,
  15 $$\begin{aligned} \lambda _0:\quad (\textbf{I} \star \textbf{I}_r)(\lambda _0) = \max _{\lambda } \{ (\textbf{I} \star \textbf{I}_r)(\lambda ). \} \end{aligned}$$
  Finally, prior to the calculation of the least squares function, we redefine
  $$I(t) \leftarrow I(t + \lambda _0)$$
  so that the offset between the two series is corrected. Indirectly, we are giving the initial phase $\phi _0$ some degree of freedom during the calibration process.
- The parameter *p* does not depend on the model, but acts *a posteriori* to the simulation, scaling the infected data series. Thus, the general solution for this optimization problem in terms of *p* is given by
  16 $$\begin{aligned}&p = \frac{ \sum _{i=1}^m I(t_i) I_r^{t_i} }{\sum _{i=1}^m I(t_i)^2 }. \end{aligned}$$
  Notice that the numerical value of *p* can be computed with this formula instead of calibrating it. Thus, we reduce the computation time (Andreu-Vilarroig et al. 2024). The parameter *p* does not affect the dynamics of the model but it depends on the $I(t_i)$ state variable, which is a outcome of the numerical simulation of model (1).

Based on the previous assumptions, the only parameter that is initially estimated is the transmission rate $\beta _0$. The calibration process consists of the following steps:

1. Initially, a sufficiently wide exploration grid for $\beta _0$ was set. In our case, we chose the range [2, 3] with 300 points, i.e., a time step of order $10^{-3}$.
2. The model is simulated, computing the reporting fraction *p* and correcting the *I*(*t*) function with the lag $\lambda _0$ after the simulation.
3. All simulations with an average percentage of new infections per season over the total population $P_{new}$ out of the 5-15% range are discarded. This percentage is computed by numerically solving the differential equation of the cumulative new infected.
  17 $$\begin{aligned} \dot{I}_{new}(t) = \beta (t) \frac{S(t) I(t)}{N(t)} + \sum _{k=1}^n \beta _k(t) \frac{R_k(t) I(t)}{N(t)} \end{aligned}$$
  and then computing the average of the percentage of new infections per season as
  18 $$\begin{aligned} P_{new}&= 100 \cdot \frac{1}{m_s} \sum _{i=1}^{m_{s} + 1} \frac{I_{new}(t_{i+1}^{s}) - I_{new}(t_i^{s})}{N} \nonumber \\&= 100 \cdot \frac{1}{m_{s}} \frac{I_{new}(t_{m_s + 1}^{s}) - I_{new}(t_1^s)}{N}, \end{aligned}$$
  where $m_s = 10$ is the number of seasons and $\{t^s_1, t^s_2,\dots ,t^s_{m_s + 1}\}$ are the inter-seasonal times, which mark the beginning (and the end) of each epidemic wave.
4. The sum of squared errors (SSE) in problem (14) is computed for the remaining simulations and the value $\beta _0^*$ that achieves the minimum SSE is returned as the optimal.

The programming of the model and the calibration method has been carried out in MATLAB. In particular, we have used the *ode45* solver for the numerical resolution of the model.

In the next section we will apply this calibration process for three different assumptions regarding the susceptibility functions defined in Eqs. (9), (10) and (11).

### Results

**Fig. 4:**
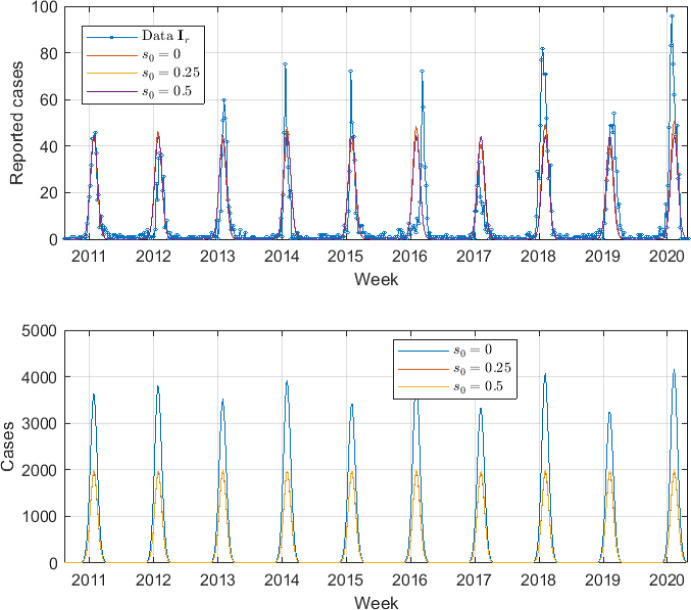
Best SIR*n* model solution. Best fit between the SIR*n* model reported infected and data series (top) and model simulation of total infected *I*(*t*) (bottom), for different susceptibility scenarios

**Fig. 5:**
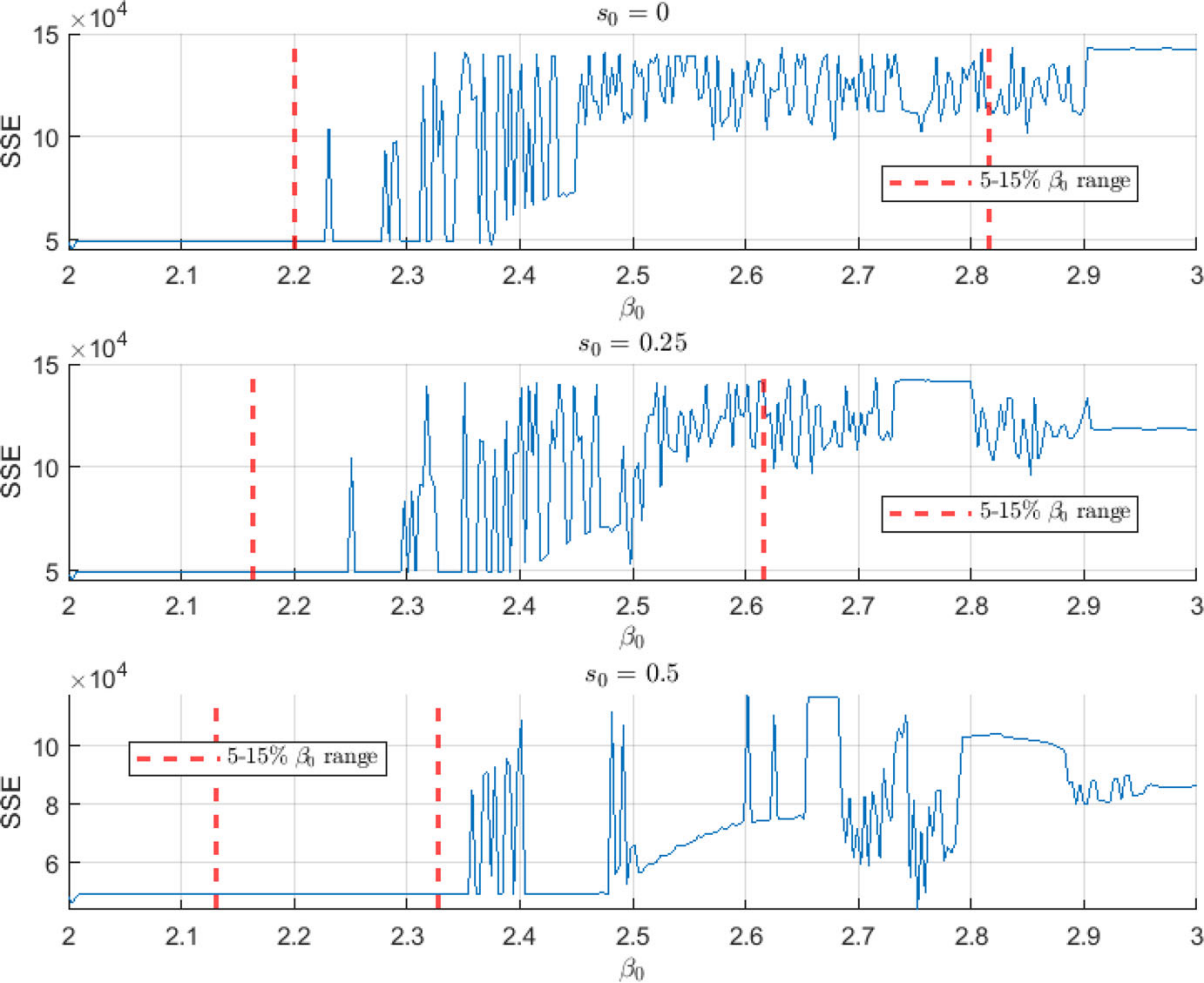
SSE error function for different $\beta _0$ parameter values and different susceptibility scenarios. The red dashed lines delimit the $\beta _0$ range that satisfies the 5-15% infected per season restriction

In this subsection, the results of the SIR*n* model fit to the seasonal flu data are shown for the case study after applying the calibration process. The calibration results are shown in Figure 4. We find the best model solution fitted to the data for the three assumed susceptibility functions $s_1(\tau )$, $s_2(\tau )$ and $s_3(\tau )$. Notice that the fitting provides very similar profiles (overlap of curves) despite the use of different susceptibility functions. Table 2, shows the $\beta _0$ value range that satisfies the 5-15% seasonal infected restriction, the mean and standard deviation (std.) of the 10% best solutions, the optimal parameter set $\theta ^*$ and the SSE value for each proposed susceptibility function. Figure 5 shows the SSE versus the different values of $\beta _0$ evaluated in the grid search. The SSE is computed by using a total of 520 data points which generates large values for the SSE.

**Table 2:** Calibration results of the seasonal SIR*n* model for different susceptibility functions $s(\tau )$: range of $\beta _0$ which satisfies the 5-15% seasonal infected restriction, mean ± std. of the 10% of best solutions $\theta$, best solution $\theta ^*$ and best SSE

**Susceptibility**	$s_1(\tau )$ ($s_0 = 0.00$)	$s_2(\tau )$ ($s_0 = 0.25$)	$s_3(\tau )$ ($s_0 = 0.50$)
**5-15% restriction** $\beta _0$ **range**	[2.200, 2.816]	[2.164, 2.615]	[2.130, 2.328]
**10% best sols.** $\theta = (\beta _0,p)$ **mean** ± **std.**	$$\begin{pmatrix} 2.249 \\ 0.0191 \end{pmatrix} \pm \begin{pmatrix} 0.0642 \\ 3.800 \cdot 10^{-3} \end{pmatrix}$$	$$\begin{pmatrix} 2.177 \\ 0.0210 \end{pmatrix} \pm \begin{pmatrix} 9.159 \cdot 10^{-3} \\ 1.165 \cdot 10^{-3} \end{pmatrix}$$	$$\begin{pmatrix} 2.139 \\ 0.0208 \end{pmatrix} \pm \begin{pmatrix} 7.225 \cdot 10^{-3} \\ 1.243 \cdot 10^{-3} \end{pmatrix}$$
**Best sol.** $\theta ^* = (\beta _0^*,p^*)$	$\left( 2.375, 0.0122\right)$	$\left( 2.164, 0.0228\right)$	$\left( 2.130, 0.0224\right)$
**Best SSE**	$4.740 \cdot 10^4$	$4.922 \cdot 10^4$	$4.919 \cdot 10^4$

As it can be observed in Figure 4, the calibration process is able to find values of $\beta _0$ and *p* parameters such that the seasonal SIR*n* model can approximately describe the real-world seasonal evolution of the influenza disease under different scenarios of susceptibility. Note that despite the forced-seasonality the peaks have different heights due to the gradual waning immunity (Goeyvaerts et al. 2015; Xiao and Moghadas 2013). Fitting a plausible mathematical model to influenza data is very challenging due to the different heights of the peaks (Goeyvaerts et al. 2015). Moreover, oftentimes the models are not identifiable. Several fundamental aspects should be remarked about these results:

1. Note that, in our model, $\mathcal {R}_0$ shows a time-dependent behaviour, since the basal infection rate $\beta (t)$ is sinusoidal. In particular, the basic reproduction number $\mathcal {R}_0(t)$ for the seasonal SIR*n* model is given by
  19 $$\begin{aligned} \mathcal {R}_0(t) = \frac{\overline{\beta }(t)}{\gamma + \mu }, \end{aligned}$$
  where
  $$\begin{aligned} \overline{\beta }(t) = \frac{1}{n + 1} \left( \beta (t) + \sum _{k=1}^n \beta _k(t) \right) , \end{aligned}$$
  is the average of all infection rates of susceptible *S* and recovered classes $R_k$ over a long period of time (Ma and Ma 2005; Goeyvaerts et al. 2015). In this case study, we consider a time unit such as $\gamma + \mu \simeq 1$, so that $\mathcal {R}_0 \simeq \overline{\beta }$. Note that $\beta _0$ is the maximum value that $\beta (t)$ can take during the season, so that $\mathcal {R}_0 \simeq \overline{\beta } < \beta _0$ (i.e., $\beta _0$ gives an upper bound of the $\mathcal {R}_0$ value). In the calibration results $\beta _0$ takes values around 2.25, 2.18 and 2.14 for the three analyzed scenarios. This agrees with what it is usually observed in other influenza mathematical modeling studies, i.e., an $\mathcal {R}_0 < 3$ (Chowell and Brauer 2009; Chowell et al. 2010; Jing et al. 2020; Nikbakht et al. 2019; Samsuzzoha et al. 2013).
2. The reporting fraction *p* is around 0.02 for the three scenarios, i.e., $2\%$ of the infected cases are urgent reported cases. Equivalently, we can say that, for every urgent reported case, there are $1/p = 50$ unreported infected cases. Note that the unreported cases include the asymptomatic cases that are able to transmit the influenza virus. Note that *p* does not represent the infection fatality ratio (IFR) nor the case fatality ratio (CFR). However, *p* must be greater than the IFR since not all the urgent reported cases end up dying. The CFR has been reported as less than $0.1\%$ and it is well-known that it is greater than the IFR (Nishiura 2017; Taubenberger and Morens 2006). Thus, the values obtained for *p* are in a realistic range.
3. The height of the peaks of years 2011-2013, 2017 and 2019 are well captured. Moreover, the time instants at which practically all the peaks occur are very close to the real ones (about ± 1-2 week of deviation with respect to the data peaks), despite the multiple transitions of individuals from the recovered *n* compartments to the infected one. This is a crucial result of the calibration of the model. Having a perfect fit of the height of the peaks is very challenging due to the introduction of new variants, age-structure, and the stochastic factors related to the infectious dynamics (Goeyvaerts et al. 2015; Allen 2008; Arenas et al. 2009).
4. The increase in the initial susceptibility level $s_0$ implies the reduction of the immunity level, making the susceptibility function closer to $s(\tau ) = 1$ (i.e., closer to the SIS model). It can be observed in Table 2 that this increase has the effect of reducing the infection rate $\beta _0$. In other words, to omit the immunity waning causes the immunity to be “transferred” to the infection rate parameter.

**Fig. 6:**
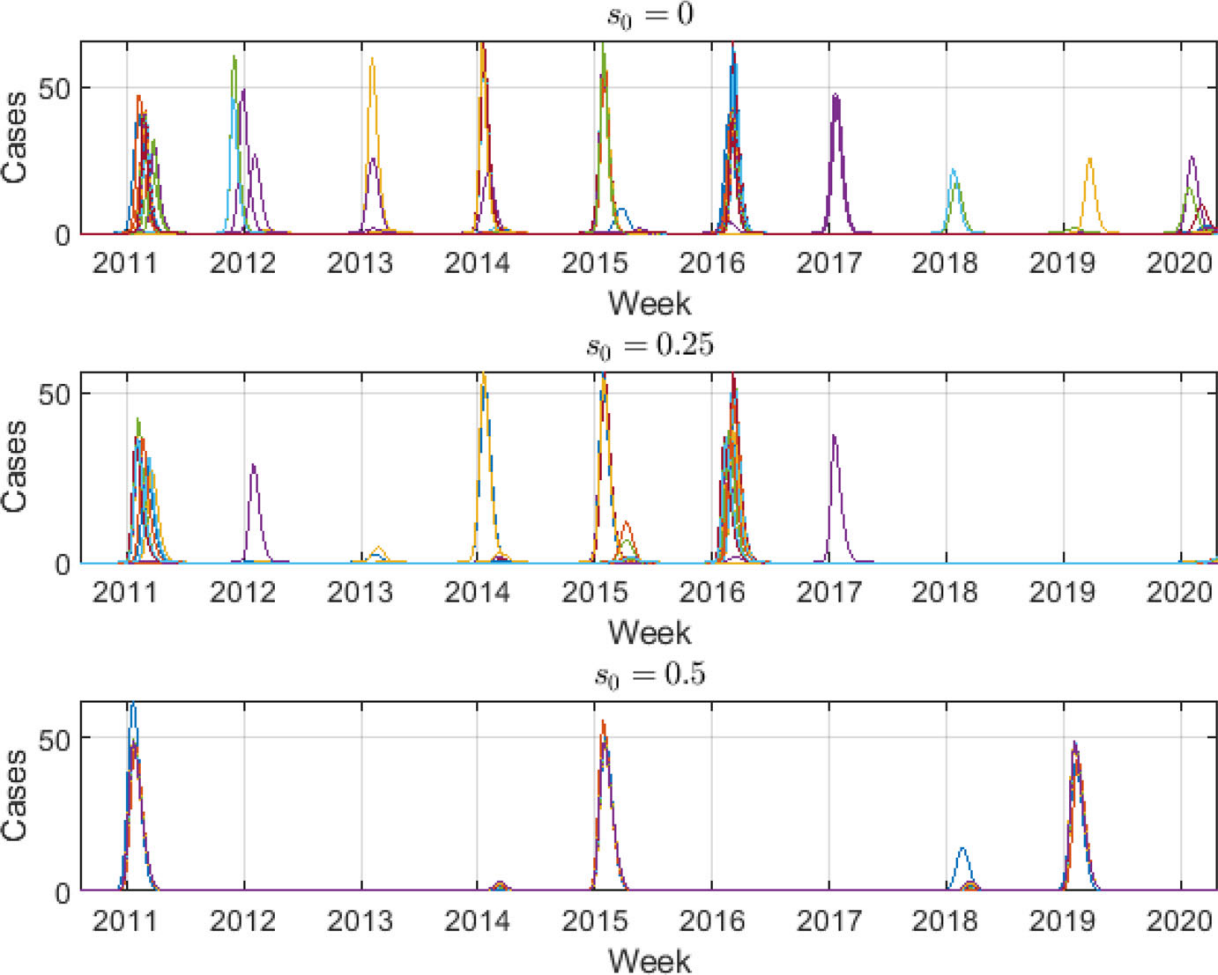
Unrealistic solutions for different susceptibility scenarios. The solutions shown are the 5% of the numerical simulations with the highest SSE

Finally, Figure 5 shows the error evolution as a function of $\beta _0$. It can be observed that the function that represents the SSE has large fluctuations and flat regions. The flat regions suggest that the optimization problem is unidentifiable (Kreutz et al. 2012; Raue et al. 2009, 2013). Since in this case we only vary the value of the parameter $\beta _0$ we are performing a practical identifiability by using the profile likelihood (Kreutz et al. 2012; Raue et al. 2009, 2013). Note that indirectly, $\lambda _0$ gives freedom to the initial phase $\phi _0$ during the calibration process. Thus, since $\beta _0$ affects the timing of the peak, it is correlated to the phase and, therefore, to $\lambda _0$. Thus, the non-identifiability of the parameters $\beta _0$, *p* and $\lambda _0$ seems reasonable because only two useful pieces of information are available for the calibration process: the series of reported infected and the 5-15% constraint of new seasonal infected (Andreu-Vilarroig et al. 2024). However, we have a set of three free parameters in the calibration: $\beta _0$, *p* and $\lambda _0$. Moreover, the seasonal constraint provides infinitely many possibilities. For the model to be identifiable, additional information should be incorporated in the calibration process (Raue et al. 2009, 2013; González-Parra et al. 2018). Nevertheless, by looking at the solutions where the SSE is large we have found that those model solutions would yield unrealistic solutions, where several season peaks disappear in the infected curves (see Figure 6). Thus, although in this problem we do not have identifiable parameters, we have found ranges for $\beta _0$ that satisfy the 5-15% constraint and that provide realistic feasible solutions. Thus, it is shown that obtaining a set of parameters’ values of the seasonal SIR*n* model such that feasible solutions are obtained is challenging (Goeyvaerts et al. 2015).

## Discussion

The mathematical modeling of the dynamics of influenza at the population level is extremely challenging due to two main factors. The first one is the seasonality nature of the influenza virus. The second one is waning immunity. Mainly, these two factors have been considered separately in epidemiological mathematical modeling. In this study, we have combined these main factors into a mathematical model. Oftentimes seasonality has been modeled by defining the influenza transmission rate as a periodic function, with higher values in winter seasons (Brauer et al. 2019; Goeyvaerts et al. 2015). The mathematical model presented in this work is in some way versatile since the time of the peak and the pattern of the epidemic wave can be modified by changing the values of some parameters of the model. This is important since different regions or countries might have slight differences in the influenza seasonal waves (Goeyvaerts et al. 2015). Different circulating influenza strains and climatic factors can create variations in the pattern of the waves.

Regarding the mathematical analysis of the proposed mathematical model it can be proven that under some conditions there are periodic solutions, but a full mathematical analysis is out of the scope of this article. The only expected steady states are the periodic solutions and a disease-free equilibrium point. Another mathematical aspect of the model is that as $n\rightarrow \infty$, the number of recovered stages in the SIR*n* model increases and then there is a continuity of recovered stages. Thus, this asymptotic model is given by an ODE-PDE system where further mathematical analysis can be done (El Khalifi and Britton 2023; Inaba 2001).

As in any modeling process, our proposed mathematical model is not a fully exhaustive modeling approach that includes all the details of the real-world. However, it results in a much more detailed model than a traditional epidemiological compartmental model such as SIR or SEIR models. The subdivision of the recovered state into *n* multiple stages, while increasing complexity, introduces richer dynamics and allows for a more realistic description of seasonal influenza by incorporating the immunity effect (El Khalifi and Britton 2023; Hethcote et al. 1981). It is important to emphasize that our proposed model considers implicitly natural cross-immunity since the decay of immunity can occur over several seasons so that the recovered population is partially protected against reinfection (Truscott et al. 2012; Xiao and Moghadas 2013). It should be noted that, while a simpler model, such as a classic SIR framework without waning immunity, could potentially achieve a similar fit to the observed data, our primary objective extended beyond purely predictive accuracy. By explicitly modeling waning immunity through the susceptibility function $s(\tau )$, we were able to disentangle its effects from the basal infection rate $\beta _0$, providing a more precise estimate of the virus transmission strength. For instance, as mentioned in Section 3.3 (point 4), omitting immunity would effectively “transfer” its impact to the $\beta _0$ parameter (by reducing it), potentially underestimating the virus infectiousness. This fact also affects to other parameters of great medical interest, such as the basic reproduction number $\mathcal {R}_0$. Thus, while a simpler models (e.g., SIR, SLIR, etc.) might predict trends similarly, our approach distinguishes the virus’s inherent infectiousness, a key insight for understanding its spread. A detailed statistical comparison with simpler models, though possible, falls outside this study’s scope, as our goal was not to optimize predictive accuracy but to provide epidemiologically meaningful parameters, especially since seasonal flu trends are already predictable with basic models once cases emerge.

To the best of our knowledge, the idea of taking into account both forced-seasonality and explicit gradual waning immunity has only been considered in a few papers, despite both factors play a crucial role in the seasonal influenza dynamics (Dafilis et al. 2014; Xiao and Moghadas 2013). Moreover, our proposed model is different than the ones presented in those works. The mathematical modeling approach proposed in this study might not be appropriate for describing the dynamics of influenza in tropical regions. In Yuan et al. (2021) it was found that a model that incorporates the effect of both humidity and temperature was able to describe the influenza epidemic patterns in Hong Kong. Their results suggested that a shorter immunity period can be used to simulate the co-circulation of influenza virus strains. Another interesting work proposed an agent-based model to describe the A/H1N1 influenza over one season. The model combines seasonality and viral mutation in an influenza pandemic and assumes that the $\mathcal {R}_0$ value varies over time by using a sinusoidal function (Shi et al. 2010).

As in any mathematical model regarding epidemics or infectious diseases, there are limitations. For instance, the constructed model does not consider the vaccination of the population explicitly. However, the model considers that since the influenza vaccination plans are implemented every year and influenza viruses have been circulating for many years then the influenza dynamics is in a steady state scenario with a seasonal behavior due to the main factors explained in this study. Thus, the vaccinated people are distributed in the different recovered subpopulations with variable immunity. The proposed mathematical modeling approach does not differentiate people with natural or induced immunity. Thus, their susceptibilities vary at the same rate over time (see Goeyvaerts et al. (2015)). We assume that the effect of this approximation is relatively small in comparison with other main factors that we have considered, such as seasonality and gradual waning immunity. The cross-immunity is considered implicitly in the susceptibility function and can be adjusted depending on the circulating influenza strains. However, the seasonal SIR*n* model does not consider explicitly subpopulations with different levels of protective cross-immunity.

Another important aspect that requires careful attention is the time after infection $\tau$. It seems plausible that an older person that have contracted the influenza virus several times might have a different immunological profile to individuals in other age groups. In the seasonal SIR*n* model there is no age-structure. Nevertheless, a potential model designed with different infectious stages depending on age might be reasonable, even though this would increase the complexity of the model (Goeyvaerts et al. 2015). There are other limitations that are more general to mathematical models based on ODEs such as the homogeneous mixing assumption (Brauer et al. 2019; Hethcote 2000). For more details about limitations of this type of mathematical models see (Goeyvaerts et al. 2015).

Recently, in El Khalifi and Britton (2023) the authors developed two mathematical models where individual immunity wanes gradually by means of linear or exponential functions. Thus, the classical SIR model is extended by including several recovered compartments. In other two works the authors have considered different levels of susceptibility of the individuals (Guiaş 2023, 2023). Nevertheless, the seasonality effect observed in influenza has not been included (Foxman et al. 2015). On the other hand, there are other works that have included seasonally-forced functions in order to deal with the seasonality of influenza, but which do not include explicit gradual waning immunity (Arenas et al. 2021; Edlund et al. 2011; Evans et al. 2005; He et al. 2013; Malek and Hoque 2024; Mahmud et al. 2023; Tanaka and Aihara 2013). There are other approaches to explain the seasonality and epidemic waves with mathematical models (González-Parra et al. 2011; Stech and Williams 1981; Yang and Jin 2021). Another aspects that could be taken into account are climate factors (Gashaw et al. 2019; González-Parra et al. 2016; Hirve et al. 2016; Moore et al. 2014; Thai et al. 2015; Yuan et al. 2021) and how they can impact the influenza seasonality. In addition, studies that provide genomic sequence analysis of the influenza viruses are useful to understand the dynamics of influenza for different discrete outbreaks (Rambaut et al. 2008).

## Conclusions

In this paper, we presented a mathematical model, the seasonal SIR*n* model, that integrates forced-seasonality and gradual waning immunity. The progressive waning immunity has been introduced into the seasonal SIR*n* model structure by considering multiple recovery stages with attenuated transmission rates by a susceptibility factor. To show the applicability and reliability of the seasonal SIR*n* model to a real-world case, we have carried out a model calibration with a data series of influenza infections reported in the 2010-2020 period at the General Hospital of Castellón de la Plana, Spain. The calibration results show that the seasonal SIR*n* model is able to approximately simulate and replicate the reported infected data. The obtained results allowed us to estimate the ranges for the numerical values of the basic reproduction number $\mathcal {R}_0$, which are in agreement with previous results from the scientific literature related to influenza. It is important to remark that the results were obtained under different different immunity decay scenarios. The model shows (through the immunity process) that the epidemic in one year is dependent on the previous years, e.g., a low epidemic in year *n* results in a higher epidemic in year $n+1$ because of lower immunity generated in year *n*. However, since the influenza virus and its variants have been present in the human population for a long time, we assumed a steady state scenario and fitted it using the seasonal SIR*n* model. We used scientific data to select different waning immunity forms (El Khalifi and Britton 2023; Ranjeva et al. 2019; Xiao and Moghadas 2013). The specific forms that we implemented for the waning immunity have not been used previously.

## References

[CR1] Abu-Raddad LJ, Ferguson NM (2004) The impact of cross-immunity, mutation and stochastic extinction on pathogen diversity. Proceedings of the Royal Society of London. Series B: Biological Sciences 271(1556):2431–2438 10.1098/rspb.2004.2877 PMC1691886 15590592

[CR2] Acedo L, Morano J-A, Villanueva R-J, Villanueva-Oller J, Díez-Domingo J (2011) Using random networks to study the dynamics of respiratory syncytial virus (RSV) in the Spanish region of Valencia. Mathematical and Computer Modelling 54(7–8):1650–1654

[CR3] Ahmed R, Oldstone MB, Palese P (2007) Protective immunity and susceptibility to infectious diseases: lessons from the 1918 influenza pandemic. Nature immunology 8(11):1188–1193 17952044 10.1038/ni1530 PMC7097533

[CR4] Allen LJ (2008) An introduction to stochastic epidemic models, in: Mathematical epidemiology, Springer, pp. 81–130

[CR5] Andreasen V, Sasaki A (2006) Shaping the phylogenetic tree of influenza by cross-immunity. Theoretical population biology 70(2):164–173 16723145 10.1016/j.tpb.2006.04.004

[CR6] Andreu-Vilarroig C, Villanueva RJ, González-Parra G (2024) Mathematical modeling for estimating influenza vaccine efficacy: A case study of the Valencian Community, Spain. Infectious Disease Modelling 9(3):744–762 10.1016/j.idm.2024.04.006 PMC11058883 38689854

[CR7] Arda B, Durusoy R, Yamazhan T, Sipahi OR, Taşbakan M, Pullukçu H, Erdem E, Ulusoy S (2011) Did the pandemic have an impact on influenza vaccination attitude? A survey among health care workers, BMC infectious diseases 11:1–8 10.1186/1471-2334-11-87 PMC3084177 21473763

[CR8] Arenas AJ, González-Parra G, De La Espriella N (2021) Nonlinear dynamics of a new seasonal epidemiological model with age-structure and nonlinear incidence rate. Computational and Applied Mathematics 40:1–27

[CR9] Arenas AJ, González-Parra G, Moraño J-A (2009) Stochastic modeling of the transmission of respiratory syncytial virus (RSV) in the region of Valencia, Spain. Biosystems 96(3):206–212 10.1016/j.biosystems.2009.01.007 19758545

[CR10] Azambuja M (2010) A parsimonious hypothesis to the cause of influenza lethality and its variations in 1918–1919 and 2009. Medical hypotheses 74(4):681–684 19962834 10.1016/j.mehy.2009.10.050 PMC7130991

[CR11] Barr J, Fearns R (2016) Chapter 2 - genetic instability of rna viruses. In: Kovalchuk I, Kovalchuk O (eds) Genome Stability. Academic Press, Boston, pp 21–35

[CR12] Barrett TJ, Patterson KC, James TM, Krüger P (2021) Impact of reduction of susceptibility to SARS-CoV-2 on epidemic dynamics in four early-seeded metropolitan regions. Scientific Reports 11(1):12213 34108496 10.1038/s41598-021-91247-7 PMC8190298

[CR13] Basile L, Oviedo de la Fuente M, Torner N, Martínez A, Jané M (2018) Real-time predictive seasonal influenza model in Catalonia. Spain. PloS one 13(3):e0193651 29513710 10.1371/journal.pone.0193651 PMC5841785

[CR14] Best K, Perelson AS (2018) Mathematical modeling of within-host Zika virus dynamics. Immunological reviews 285(1):81–96 30129207 10.1111/imr.12687 PMC6107313

[CR15] Bhattacharya S, Adler FR (2012) A time since recovery model with varying rates of loss of immunity. Bulletin of mathematical biology 74:2810–2819 23097124 10.1007/s11538-012-9780-7

[CR16] Brauer F, Castillo-Chavez C, Castillo-Chavez C (2012) Mathematical models in population biology and epidemiology, vol 2. Springer, New York

[CR17] Brauer F, Castillo-Chavez C, Feng Z, Brauer F, Castillo-Chavez C, Feng Z (2019) Models for influenza. Mathematical Models in Epidemiology 311–350

[CR18] Brugger J, Althaus CL (2020) Transmission of and susceptibility to seasonal influenza in Switzerland from 2003 to 2015. Epidemics 30:100373 31635972 10.1016/j.epidem.2019.100373

[CR19] Castillo-Rodríguez L, Malo-Sánchez D, Díaz-Jiménez D, García-Velásquez I, Pulido P, Castañeda-Orjuela C (2022) Economic costs of severe seasonal influenza in Colombia, 2017–2019: A multi-center analysis. Plos One 17(6):e0270086 35714144 10.1371/journal.pone.0270086 PMC9205505

[CR20] Centers for Disease Control and Prevention (CDC), Influenza (Flu), https://www.cdc.gov/flu/about/disease/spread.htm, [Accessed: 2023-10-27] (2023)

[CR21] Chan MC, Wang MH, Chen Z, Hui DS, Kwok AK, Yeung AC, Liu KM, Yeoh YK, Lee N, Chan PK (2018) Frequent genetic mismatch between vaccine strains and circulating seasonal influenza viruses, Hong Kong, China, 1996–2012. Emerging Infectious Diseases 24(10):1825 10.3201/eid2410.180652 PMC6154132 30226188

[CR22] Chen Z, Bancej C, Lee L, Champredon D (2022) Antigenic drift and epidemiological severity of seasonal influenza in Canada. Scientific Reports 12(1):15625 36115880 10.1038/s41598-022-19996-7 PMC9482630

[CR23] Chowell G (2017) Fitting dynamic models to epidemic outbreaks with quantified uncertainty: A primer for parameter uncertainty, identifiability, and forecasts. Infectious Disease Modelling 2(3):379–398 29250607 10.1016/j.idm.2017.08.001 PMC5726591

[CR24] Chowell G, Brauer F (2009) The basic reproduction number of infectious diseases: computation and estimation using compartmental epidemic models. Mathematical and statistical estimation approaches in epidemiology 1–30

[CR25] Chowell G, Viboud C, Simonsen L, Miller M, Alonso WJ (2010) The reproduction number of seasonal influenza epidemics in Brazil, 1996–2006. Proceedings of the Royal Society B: Biological Sciences 277(1689):1857–1866 10.1098/rspb.2009.1897 PMC2871867 20150218

[CR26] Cobey S, Hensley SE (2017) Immune history and influenza virus susceptibility. Current Opinion in Virology 22:105–111 28088686 10.1016/j.coviro.2016.12.004 PMC5467731

[CR27] Combadière B, Sibéril S, Duffy D (2010) Keeping the memory of influenza viruses. Pathologie Biologie 58(2):e79–e86 20303671 10.1016/j.patbio.2010.01.010

[CR28] Cox R, Brokstad K, Ogra P (2004) Influenza virus: immunity and vaccination strategies. comparison of the immune response to inactivated and live, attenuated influenza vaccines. Scandinavian Journal of immunology 59(1):1–15 10.1111/j.0300-9475.2004.01382.x 14723616

[CR29] Dafilis MP, Frascoli F, McVernon J, Heffernan JM, McCaw JM (2014) The dynamical consequences of seasonal forcing, immune boosting and demographic change in a model of disease transmission. Journal of Theoretical Biology 361:124–132 25106793 10.1016/j.jtbi.2014.07.028

[CR30] Dalziel BD, Kissler S, Gog JR, Viboud C, Bjørnstad ON, Metcalf CJE, Grenfell BT (2018) Urbanization and humidity shape the intensity of influenza epidemics in US cities. Science 362(6410):75–79 30287659 10.1126/science.aat6030 PMC6510303

[CR31] De Cezaro A, Gomes ACFN, Marques JC (2023) Immunological memory improves the long-term cross-immunity: An influenza case study. Ciência e Natura 45(esp. 3):e73999–e73999

[CR32] Departamento de Seguridad Nacional, Gripe: Evolución de la difusión geográfica en España, https://www.dsn.gob.es/es/actualidad/sala-prensa/gripe-estacional-y-su-actividad, [Accessed: 2023-11-3] (2023)

[CR33] Dobrovolny HM, Reddy MB, Kamal MA, Rayner CR, Beauchemin CA (2013) Assessing mathematical models of influenza infections using features of the immune response. PloS one 8(2):e57088 23468916 10.1371/journal.pone.0057088 PMC3585335

[CR34] Doherty PC, Turner SJ, Webby RG, Thomas PG (2006) Influenza and the challenge for immunology. Nature immunology 7(5):449–455 16622432 10.1038/ni1343

[CR35] Edlund S, Kaufman J, Lessler J, Douglas J, Bromberg M, Kaufman Z, Bassal R, Chodick G, Marom R, Shalev V et al (2011) Comparing three basic models for seasonal influenza. Epidemics 3(3–4):135–142 22094336 10.1016/j.epidem.2011.04.002

[CR36] Ehrhardt M, Gašper J, Kilianová S (2019) SIR-based mathematical modeling of infectious diseases with vaccination and waning immunity. Journal of Computational Science 37:101027

[CR37] El Khalifi M, Britton T (2023) Extending susceptible-infectious-recovered-susceptible epidemics to allow for gradual waning of immunity. Journal of the Royal Society Interface 20(206):20230042 37700711 10.1098/rsif.2023.0042 PMC10498349

[CR38] Endo A, Uchida M, Hayashi N, Liu Y, Atkins KE, Kucharski AJ, Funk S (2021) Within and between classroom transmission patterns of seasonal influenza among primary school students in Matsumoto city, Japan. Proceedings of the National Academy of Sciences 118(46):e2112605118 10.1073/pnas.2112605118 PMC8609560 34753823

[CR39] Epstein SL, Price GE (2010) Cross-protective immunity to influenza A viruses. Expert review of vaccines 9(11):1325–1341 21087110 10.1586/erv.10.123

[CR40] Evans ND, White LJ, Chapman MJ, Godfrey KR, Chappell MJ (2005) The structural identifiability of the susceptible infected recovered model with seasonal forcing. Mathematical biosciences 194(2):175–197 15854675 10.1016/j.mbs.2004.10.011

[CR41] Ewing A, Lee EC, Viboud C, Bansal S (2017) Contact, travel, and transmission: The impact of winter holidays on influenza dynamics in the United States. The Journal of infectious diseases 215(5):732–739 28031259 10.1093/infdis/jiw642 PMC5853779

[CR42] Foxman EF, Storer JA, Fitzgerald ME, Wasik BR, Hou L, Zhao H, Turner PE, Pyle AM, Iwasaki A (2015) Temperature-dependent innate defense against the common cold virus limits viral replication at warm temperature in mouse airway cells. Proceedings of the National Academy of Sciences 112(3):827–832 10.1073/pnas.1411030112 PMC4311828 25561542

[CR43] Foxman EF, Storer JA, Vanaja K, Levchenko A, Iwasaki A (2016) Two interferon-independent double-stranded RNA-induced host defense strategies suppress the common cold virus at warm temperature. Proceedings of the National Academy of Sciences 113(30):8496–8501 10.1073/pnas.1601942113 PMC4968739 27402752

[CR44] Furuse Y, Oshitani H (2016) Mechanisms of replacement of circulating viruses by seasonal and pandemic influenza A viruses. International Journal of Infectious Diseases 51:6–14 27569827 10.1016/j.ijid.2016.08.012

[CR45] Gabrick EC, Brugnago EL, de Souza SL, Iarosz KC, Szezech JD, Viana RL, Caldas IL, Batista AM, Kurths J (2024) Impact of periodic vaccination in seirs seasonal model, Chaos: An Interdisciplinary Journal of Nonlinear Science 34(1) 10.1063/5.0169834 38271628

[CR46] Gagnon A, Acosta JE, Madrenas J, Miller MS (2015) Is antigenic sin always “original?’’ Re-examining the evidence regarding circulation of a human H1 influenza virus immediately prior to the 1918 Spanish flu. PLoS pathogens 11(3):e1004615 25742615 10.1371/journal.ppat.1004615 PMC4351064

[CR47] Gagnon A, Miller MS, Hallman SA, Bourbeau R, Herring DA, Earn DJ, Madrenas J (2013) Age-specific mortality during the 1918 influenza pandemic: unravelling the mystery of high young adult mortality. PloS one 8(8):e69586 23940526 10.1371/journal.pone.0069586 PMC3734171

[CR48] Gashaw KW, Kassa SM, Ouifki R (2019) Climate-dependent malaria disease transmission model and its analysis. International Journal of Biomathematics 12(08):1950087

[CR49] Gibson E, Begum N, Martinón-Torres F, Safadi MA, Sackeyfio A, Hackett J, Rajaram S (2016) Cost-effectiveness analysis of the direct and indirect impact of intranasal live attenuated influenza vaccination strategies in children: alternative country profiles. Journal of Market Access & Health Policy 4(1):31205 10.3402/jmahp.v4.31205 PMC4928186 27429720

[CR50] Goeyvaerts N, Willem L, Van Kerckhove K, Vandendijck Y, Hanquet G, Beutels P, Hens N (2015) Estimating dynamic transmission model parameters for seasonal influenza by fitting to age and season-specific influenza-like illness incidence. Epidemics 13:1–9 26616037 10.1016/j.epidem.2015.04.002

[CR51] González-Parra G, Arenas AJ, Aranda DF, Segovia L (2011) Modeling the epidemic waves of AH1N1/09 influenza around the world. Spatial and spatio-temporal epidemiology 2(4):219–226 22748221 10.1016/j.sste.2011.05.002

[CR52] González-Parra G, De Ridder F, Huntjens D, Roymans D, Ispas G, Dobrovolny HM (2018) A comparison of RSV and influenza in vitro kinetic parameters reveals differences in infecting time. PloS one 13(2):e0192645 29420667 10.1371/journal.pone.0192645 PMC5805318

[CR53] González-Parra G, Dobrovolny HM, Aranda DF, Chen-Charpentier B, Rojas RAG (2018) Quantifying rotavirus kinetics in the REH tumor cell line using in vitro data. Virus research 244:53–63 29109019 10.1016/j.virusres.2017.09.023

[CR54] González-Parra G, Querales JF, Aranda D (2016) Prediction of the respiratory syncitial virus epidemic using climate variables in Bogotá. DC, Biomédica 36(3):378–389 27869386 10.7705/biomedica.v36i3.2763

[CR55] Grassly NC, Fraser C (2006) Seasonal infectious disease epidemiology. Proceedings of the Royal Society B: Biological Sciences 273(1600):2541–2550 10.1098/rspb.2006.3604 PMC1634916 16959647

[CR56] Greiff DR, Patterson-Robert A, Blyth CC, Glass K, Moore HC (2021) Epidemiology and seasonality of human parainfluenza serotypes 1–3 in Australian children. Influenza and Other Respiratory Viruses 15(5):661–669 33491337 10.1111/irv.12838 PMC8404051

[CR57] Guiaş F (2023) Epidemic models with several levels of immunity. Quantitative Demography and Health Estimates: Healthy Life Expectancy. Templates for Direct Estimates from Life Tables and other Applications, Springer, Berlin, Heidelberg, pp 163–174

[CR58] Guiaş F (2023) Equilibrium solutions of a modified SIR model with vaccination and several levels of immunity. WSEAS Transactions on Systems and Control 18:550–560

[CR59] He D, Dushoff J, Eftimie R, Earn DJ (2013) Patterns of spread of influenza A in Canada. Proceedings of the Royal Society B: Biological Sciences 280(1770):20131174 10.1098/rspb.2013.1174 PMC3779324 24026815

[CR60] Hethcote HW (2000) The mathematics of infectious diseases. SIAM review 42(4):599–653

[CR61] Hethcote HW, Stech HW, Van Den Driessche P (1981) Nonlinear oscillations in epidemic models. SIAM Journal on Applied Mathematics 40(1):1–9

[CR62] Hill EM, Petrou S, de Lusignan S, Yonova I, Keeling MJ (2019) Seasonal influenza: Modelling approaches to capture immunity propagation. PLoS computational biology 15(10):e1007096 31658250 10.1371/journal.pcbi.1007096 PMC6837557

[CR63] Hirve S, Newman LP, Paget J, Azziz-Baumgartner E, Fitzner J, Bhat N, Vandemaele K, Zhang W (2016) Influenza seasonality in the tropics and subtropics-when to vaccinate? PloS one 11(4):e0153003 27119988 10.1371/journal.pone.0153003 PMC4847850

[CR64] Ho SH, He D, Eftimie R (2019) Mathematical models of transmission dynamics and vaccine strategies in Hong Kong during the 2017–2018 winter influenza season. Journal of theoretical biology 476:74–94 31128142 10.1016/j.jtbi.2019.05.013

[CR65] Huang D, Taha MS, Nocera AL, Workman AD, Amiji MM, Bleier BS (2023) Cold exposure impairs extracellular vesicle swarm-mediated nasal antiviral immunity. Journal of Allergy and Clinical Immunology 151(2):509–525 36494212 10.1016/j.jaci.2022.09.037 PMC13380700

[CR66] Imai C, Toizumi M, Hall L, Lambert S, Halton K, Merollini K (2018) A systematic review and meta-analysis of the direct epidemiological and economic effects of seasonal influenza vaccination on healthcare workers. PloS one 13(6):e0198685 29879206 10.1371/journal.pone.0198685 PMC5991711

[CR67] Inaba H (2001) Kermack and McKendrick revisited: the variable susceptibility model for infectious diseases. Japan journal of industrial and applied mathematics 18:273–292

[CR68] Instituto Nacional de Estadística (INE), Indicadores demográficos básicos, https://www.ine.es/, [Accessed: 2023-10-28] (2023)

[CR69] Jing S-L, Huo H-F, Xiang H (2020) Modeling the effects of meteorological factors and unreported cases on seasonal influenza outbreaks in Gansu province, China. Bulletin of Mathematical Biology 82:1–36 10.1007/s11538-020-00747-6 32533498

[CR70] Kenah E, Chao DL, Matrajt L, Halloran ME, Longini IM Jr (2011) The global transmission and control of influenza. PloS one 6(5):e19515 21573121 10.1371/journal.pone.0019515 PMC3089626

[CR71] Kim H, Webster RG, Webby RJ (2018) Influenza virus: dealing with a drifting and shifting pathogen. Viral immunology 31(2):174–183 29373086 10.1089/vim.2017.0141

[CR72] Krammer F (2019) The human antibody response to influenza A virus infection and vaccination. Nature Reviews Immunology 19(6):383–397 10.1038/s41577-019-0143-6 30837674

[CR73] Kreutz C, Raue A, Timmer J (2012) Likelihood based observability analysis and confidence intervals for predictions of dynamic models. BMC Systems Biology 6(1):1–9 22947028 10.1186/1752-0509-6-120 PMC3490710

[CR74] Kubo T, Morita H, Sugita K, Akdis CA (2017) Chapter 1 - Introduction to mechanisms of allergic diseases. In: O’Hehir RE, Holgate ST, Sheikh A (eds) Middleton’s Allergy Essentials. Elsevier, London, pp 1–27

[CR75] Lai DZ, Gog JR (2024) Waning immunity can drive repeated waves of infections. Mathematical Biosciences and Engineering 21(2):1979–2003 38454671 10.3934/mbe.2024088

[CR76] Li C, Hatta M, Burke DF, Ping J, Zhang Y, Ozawa M, Taft AS, Das SC, Hanson AP, Song J et al (2016) Selection of antigenically advanced variants of seasonal influenza viruses. Nature microbiology 1(6):1–10 10.1038/nmicrobiol.2016.58 PMC5087998 27572841

[CR77] Liu V, Walker S (2023) Testing for genetic mutation of seasonal influenza virus. Journal of Applied Statistics 50(1):1–18 36530774 10.1080/02664763.2021.1978955 PMC9754041

[CR78] Lloyd AL (2001) Realistic distributions of infectious periods in epidemic models: changing patterns of persistence and dynamics. Theoretical population biology 60(1):59–71 11589638 10.1006/tpbi.2001.1525

[CR79] Lofgren E, Fefferman NH, Naumov YN, Gorski J, Naumova EN (2007) Influenza seasonality: underlying causes and modeling theories. Journal of virology 81(11):5429–5436 17182688 10.1128/JVI.01680-06 PMC1900246

[CR80] Lowen AC, Steel J (2014) Roles of humidity and temperature in shaping influenza seasonality. Journal of virology 88(14):7692–7695 24789791 10.1128/JVI.03544-13 PMC4097773

[CR81] Ma J, Ma Z (2005) Epidemic threshold conditions for seasonally forced seir models. Mathematical Biosciences & Engineering 3(1):161–172 10.3934/mbe.2006.3.161 20361816

[CR82] Magal P, Noussair A, Webb G, Wu Y (2020) Modeling epidemic outbreaks in geographical regions: Seasonal influenza in Puerto Rico. Discret. Contin. Dyn. Syst. Ser. S 13:3535

[CR83] Mahmud AS, Martinez PP, Baker RE (2023) The impact of current and future climates on spatiotemporal dynamics of influenza in a tropical setting. PNAS Nexus 2(9):pgad307 10.1093/pnasnexus/pgad307 PMC11089418 38741656

[CR84] Malek A, Hoque A (2024) Mathematical modeling of the infectious spread and outbreak dynamics of avian influenza with seasonality transmission for chicken farms. Comparative Immunology, Microbiology and Infectious Diseases 104:102108 38070401 10.1016/j.cimid.2023.102108

[CR85] McAuley JL, Kedzierska K, Brown LE, Shanks GD (2015) Host immunological factors enhancing mortality of young adults during the 1918 influenza pandemic. Frontiers in immunology 6:419 26347742 10.3389/fimmu.2015.00419 PMC4541073

[CR86] Moore HC, Jacoby P, Hogan AB, Blyth CC, Mercer GN (2014) Modelling the seasonal epidemics of respiratory syncytial virus in young children. PloS one 9(6):e100422 24968133 10.1371/journal.pone.0100422 PMC4072624

[CR87] Moorthy M, Castronovo D, Abraham A, Bhattacharyya S, Gradus S, Gorski J, Naumov YN, Fefferman NH, Naumova EN (2012) Deviations in influenza seasonality: odd coincidence or obscure consequence? Clinical microbiology and infection 18(10):955–962 22958213 10.1111/j.1469-0691.2012.03959.x PMC3442949

[CR88] Natoli G, Ostuni R (2019) Adaptation and memory in immune responses. Nature immunology 20(7):783–792 31213714 10.1038/s41590-019-0399-9

[CR89] Neher RA, Bedford T, Daniels RS, Russell CA, Shraiman BI (2016) Prediction, dynamics, and visualization of antigenic phenotypes of seasonal influenza viruses. Proceedings of the National Academy of Sciences 113(12):E1701–E1709 10.1073/pnas.1525578113 PMC4812706 26951657

[CR90] Neumann G, Kawaoka Y (2022) Seasonality of influenza and other respiratory viruses. EMBO Molecular Medicine 14(4):e15352 35157360 10.15252/emmm.202115352 PMC8988196

[CR91] Nikbakht R, Baneshi MR, Bahrampour A, Hosseinnataj A (2019) Comparison of methods to estimate basic reproduction number (R0) of influenza, using Canada 2009 and 2017–18 A (H1N1) data. Journal of Research in Medical Sciences 24(1):67 31523253 10.4103/jrms.JRMS_888_18 PMC6670001

[CR92] Nishiura H (2017) Real-time estimation of the case fatality ratio and risk factors of death. Handbook of statistics, vol 36. Elsevier, Netherlands, pp 167–174

[CR93] Noymer A, Garenne M (2000) The 1918 influenza epidemic’s effects on sex differentials in mortality in the United States. Population and development review 26(3):565–581 19530360 10.1111/j.1728-4457.2000.00565.x PMC2740912

[CR94] Olmos Liceaga D, Nunes SF, Saenz RA (2023) Ex vivo experiments shed light on the innate immune response from influenza virus. Bulletin of Mathematical Biology 85(11):115 37833614 10.1007/s11538-023-01217-5

[CR95] Omori R, Sasaki A (2013) Timing of the emergence of new successful viral strains in seasonal influenza. Journal of theoretical biology 329:32–38 23567650 10.1016/j.jtbi.2013.03.027

[CR96] O’Neill PD, Becker NG (2001) Inference for an epidemic when susceptibility varies. Biostatistics 2(1):99–108 12933559 10.1093/biostatistics/2.1.99

[CR97] Papadopoulos NG, Sanderson G, Hunter J, Johnston SL (1999) Rhinoviruses replicate effectively at lower airway temperatures. Journal of medical virology 58(1):100–104 10223554 10.1002/(sici)1096-9071(199905)58:1<100::aid-jmv16>3.0.co;2-d

[CR98] Patel MM, York IA, Monto AS, Thompson MG, Fry AM (2021) Immune-mediated attenuation of influenza illness after infection: opportunities and challenges. The Lancet Microbe 2(12):e715–e725 35544110 10.1016/S2666-5247(21)00180-4

[CR99] Pitman R, Nagy L, Sculpher M (2013) Cost-effectiveness of childhood influenza vaccination in England and Wales: results from a dynamic transmission model. Vaccine 31(6):927–942 23246550 10.1016/j.vaccine.2012.12.010

[CR100] Putri WC, Muscatello DJ, Stockwell MS, Newall AT (2018) Economic burden of seasonal influenza in the United States. Vaccine 36(27):3960–3966 29801998 10.1016/j.vaccine.2018.05.057

[CR101] Rabiner LR, Gold B (1975) Theory and application of digital signal processing. Prentice-Hall, Englewood Cliffs

[CR102] Rambaut A, Pybus OG, Nelson MI, Viboud C, Taubenberger JK, Holmes EC (2008) The genomic and epidemiological dynamics of human influenza a virus. Nature 453(7195):615–619 18418375 10.1038/nature06945 PMC2441973

[CR103] Ranjeva S, Subramanian R, Fang VJ, Leung GM, Ip DK, Perera RA, Peiris JM, Cowling BJ, Cobey S (2019) Age-specific differences in the dynamics of protective immunity to influenza. Nature Communications 10(1):1660 10.1038/s41467-019-09652-6 PMC6458119 30971703

[CR104] Ratajczak W, Niedźwiedzka-Rystwej P, Tokarz-Deptuła B, Deptuła W (2018) Immunological memory cells. Central European Journal of Immunology 43(2):194–203 30135633 10.5114/ceji.2018.77390 PMC6102609

[CR105] Raue A, Kreutz C, Maiwald T, Bachmann J, Schilling M, Klingmüller U, Timmer J (2009) Structural and practical identifiability analysis of partially observed dynamical models by exploiting the profile likelihood. Bioinformatics 25(15):1923–1929 19505944 10.1093/bioinformatics/btp358

[CR106] Raue A, Kreutz C, Theis FJ, Timmer J (2013) Joining forces of Bayesian and frequentist methodology: a study for inference in the presence of non-identifiability, Philosophical Transactions of the Royal Society A: Mathematical. Physical and Engineering Sciences 371(1984):20110544 10.1098/rsta.2011.0544 23277602

[CR107] Russell CA, Jones TC, Barr IG, Cox NJ, Garten RJ, Gregory V, Gust ID, Hampson AW, Hay AJ, Hurt AC et al (2008) Influenza vaccine strain selection and recent studies on the global migration of seasonal influenza viruses. Vaccine 26:D31–D34 19230156 10.1016/j.vaccine.2008.07.078

[CR108] Samsuzzoha M, Singh M, Lucy D (2013) Uncertainty and sensitivity analysis of the basic reproduction number of a vaccinated epidemic model of influenza. Applied Mathematical Modelling 37(3):903–915

[CR109] Sara B, Omar Z, Abdessamad T, Mostafa R, Hanane F (2020) Parameters’ estimation, sensitivity analysis and model uncertainty for an influenza a mathematical model: case of Morocco, Commun. Math. Biol. Neurosci. 2020 Article–ID

[CR110] Shi P, Keskinocak P, Swann J, Lee B (2010) Modelling seasonality and viral mutation to predict the course of an influenza pandemic. Epidemiology & Infection 138(10):1472–1481 20158932 10.1017/S0950268810000300 PMC3779923

[CR111] Shubin M, Lebedev A, Lyytikäinen O, Auranen K (2016) Revealing the true incidence of pandemic A (H1N1) pdm09 influenza in Finland during the first two seasons–an analysis based on a dynamic transmission model. PLoS computational biology 12(3):e1004803 27010206 10.1371/journal.pcbi.1004803 PMC4807082

[CR112] Stöhr K (2002) Influenza–who cares. The Lancet infectious diseases 2(9):517 12206966 10.1016/s1473-3099(02)00366-3

[CR113] Stech H, Williams M (1981) Stability in a class of cyclic epidemic models with delay. Journal of Mathematical Biology 11:95–103

[CR114] Takeuchi S, Kuroda Y (2010) Predicting spread of new pandemic swine-origin influenza A (H1N1) in local mid-size city: evaluation of hospital bed shortage and effectiveness of vaccination, Nihon Eiseigaku zasshi. Japanese Journal of Hygiene 65(1):48–52 20134108 10.1265/jjh.65.48

[CR115] Tamerius J, Nelson MI, Zhou SZ, Viboud C, Miller MA, Alonso WJ (2011) Global influenza seasonality: reconciling patterns across temperate and tropical regions. Environmental health perspectives 119(4):439–445 21097384 10.1289/ehp.1002383 PMC3080923

[CR116] Tanaka G, Aihara K (2013) Effects of seasonal variation patterns on recurrent outbreaks in epidemic models. Journal of theoretical biology 317:87–95 23041433 10.1016/j.jtbi.2012.09.038

[CR117] Tang JW-T, Loh TP (2016) Influenza seasonality. Current Treatment Options in Infectious Diseases 8:343–367

[CR118] Taubenberger JK, Morens DM (2006) 1918 Influenza: the mother of all pandemics. Revista Biomedica 17(1):69–79 10.3201/eid1201.050979 PMC3291398 16494711

[CR119] Teixeira CA, Mendes L, Ruano MG, Pereira WC (2017) A method for sub-sample computation of time displacements between discrete signals based only on discrete correlation sequences. Biomedical Signal Processing and Control 31:560–568

[CR120] Thai PQ, Choisy M, Duong TN, Thiem VD, Yen NT, Hien NT, Weiss DJ, Boni MF, Horby P (2015) Seasonality of absolute humidity explains seasonality of influenza-like illness in Vietnam. Epidemics 13:65–73 26616043 10.1016/j.epidem.2015.06.002

[CR121] Tokars JI, Olsen SJ, Reed C (2018) Seasonal incidence of symptomatic influenza in the United States. Clinical Infectious Diseases 66(10):1511–1518 29206909 10.1093/cid/cix1060 PMC5934309

[CR122] Towers S, Feng Z (2009) Pandemic H1N1 influenza: predicting the course of a pandemic and assessing the efficacy of the planned vaccination programme in the United States. Eurosurveillance 14(41):19358 19883540

[CR123] Tracht SM, Del Valle SY, Hyman JM (2010) Mathematical modeling of the effectiveness of facemasks in reducing the spread of novel influenza A (H1N1). PloS one 5(2):e9018 20161764 10.1371/journal.pone.0009018 PMC2818714

[CR124] Truscott J, Fraser C, Cauchemez S, Meeyai A, Hinsley W, Donnelly CA, Ghani A, Ferguson N (2012) Essential epidemiological mechanisms underpinning the transmission dynamics of seasonal influenza. Journal of The Royal Society Interface 9(67):304–312 21715400 10.1098/rsif.2011.0309 PMC3243394

[CR125] Webster RG, Govorkova EA (2014) Continuing challenges in influenza. Annals of the New York Academy of Sciences 1323(1):115–139 24891213 10.1111/nyas.12462 PMC4159436

[CR126] World Health Organization (WHO) (2024) Influenza (seasonal), https://www.who.int/news-room/fact-sheets/detail/influenza-(seasonal), [Accessed: 2024-03-01]

[CR127] Xiao Y, Moghadas SM (2013) Impact of viral drift on vaccination dynamics and patterns of seasonal influenza. BMC Infectious Diseases 13:1–11 24330575 10.1186/1471-2334-13-589 PMC4028866

[CR128] Yang H, Jin Z (2021) A stochastic model explains the periodicity phenomenon of influenza on network. Scientific reports 11(1):20996 34697349 10.1038/s41598-021-00260-3 PMC8546073

[CR129] Yuan H, Kramer SC, Lau EH, Cowling BJ, Yang W (2021) Modeling influenza seasonality in the tropics and subtropics. PLoS Computational Biology 17(6):e1009050 34106917 10.1371/journal.pcbi.1009050 PMC8216520

[CR130] Zhang X-S (2015) Strain interactions as a mechanism for dominant strain alternation and incidence oscillation in infectious diseases: seasonal influenza as a case study. PloS one 10(11):e0142170 26562668 10.1371/journal.pone.0142170 PMC4642928

